# Biofabrication of a 3D human skeletal muscle microenvironment to study the early steps of fibrosis

**DOI:** 10.1016/j.mtbio.2025.102386

**Published:** 2025-10-15

**Authors:** R. Francescato, M. Ishmaku, G. Talò, M. Francese, L. Cascione, V. Martini, M. Uguccioni, M. Moretti, S. Bersini

**Affiliations:** a Regenerative Medicine Division, Institute for Translational Research (IRT), Faculty of Biomedical Sciences, Università della Svizzera italiana (USI) and Ente Ospedaliero Cantonale (EOC), Switzerland; b Servizio di Ortopedia e Traumatologia, Ente Ospedaliero Cantonale, Lugano, Switzerland; c Euler Institute, Faculty of Biomedical Sciences, Università della Svizzera Italiana, Lugano, Switzerland; d Cell and Tissue Engineering Laboratory, IRCCS Ospedale Galeazzi – Sant’Ambrogio, Milano, Italy; e Department of Chemistry, Materials and Chemical Engineering ‘Giulio Natta’, Politecnico di Milano, Milano, Italy; f Lymphoma Genomics, Institute of Oncology Research, Bellinzona, Switzerland; g Institute for Research in Biomedicine (IRB), Università della Svizzera Italiana, Bellinzona, Switzerland

## Abstract

**Figure:**
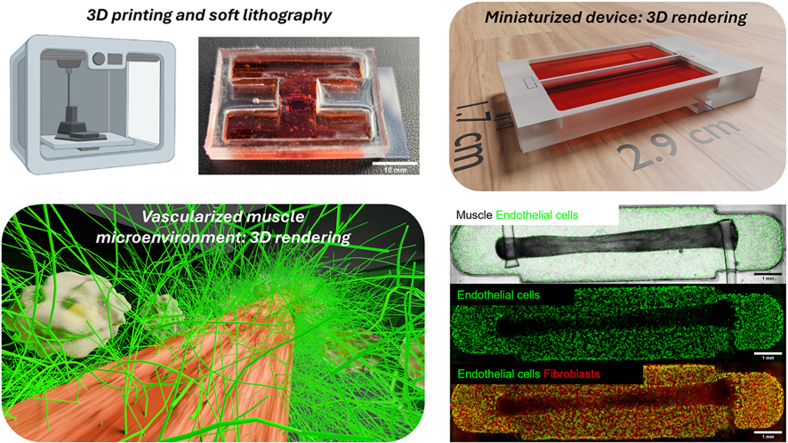

## 1 Introduction

Skeletal muscle fibrosis is a characteristic feature observed in myopathies, traumatic injuries [1] and muscular dystrophies [2] posing a significant healthcare and economical burden [[3], [4], [5]]. Fibrosis arises due to a complex interplay of molecular and mechanical alterations within the tissue, resulting in the progressive and uncontrolled deposition of extracellular matrix (ECM), finally leading to muscle weakness, reduced tissue elasticity and atrophy. This process leads to decreased mobility due to the continual loss of functional myofibers and their replacement with non-contractile cells, coupled with significant tissue scarring [6], which also impairs the delivery of treatments [7]. Thus, muscle fibrosis poses a substantial barrier to the restoration of tissue functionality and represents a significant challenge in musculoskeletal research.

Recent studies have demonstrated that beyond fibroblasts other muscle-resident cells (e.g. endothelial cells (ECs), macrophages, fibro adipogenic progenitors) can impact the onset and progression of fibrosis. Most importantly, ECs can undergo phenotypic changes through an endothelial-mesenchymal transition (EndoMT), hence acquiring a myofibroblastic phenotype (e.g. alpha-smooth muscle actin - αSMA, N-cadherin expression) [8,9]. While EndoMT has been implicated in fibrosis of organs such as the lung, kidney, and heart, its contribution to skeletal muscle fibrosis remains poorly understood and largely unexplored. These myofibroblasts play a pivotal role in fibrosis by overproducing ECM (e.g. collagen type I, fibronectin) and secreting pro-fibrotic molecules such as proteolytic enzymes (e.g. matrix metalloproteinases - MMPs, tissue inhibitors of metalloproteinases - TIMPs), angiogenic factors (e.g. vascular endothelial growth factor - VEGF, hepatocyte growth factor/scatter factor - HGF/SF), and fibrogenic cytokines (e.g. Transforming growth factor beta - TGFβ isoforms) [10,11].

Overall, this complex scenario has hindered the development of effective therapeutic strategies, partially due to the absence of pre-clinical models faithfully recapitulating human muscle patho-physiology. Traditionally, fibrosis has been studied through animal models that cannot fully mimic the human counterpart due to genetic, molecular and cellular differences [12,13]. For instance, the mdx mouse commonly used for pre-clinical studies on Duchenne Muscular Dystrophy (DMD) cannot recapitulate the evolution and clinical outcome of fibrosis observed in humans (e.g. almost normal lifespan, only minor clinical dysfunctions) [14]. Moreover, screening systems based on animal models often struggle to discern tissue-specific responses from compensatory effects [15] and are inherently low-throughput, making them expensive and time-consuming. On the other side, 2D cultures of human skeletal muscle cells have been widely employed for drug testing and disease modeling [16]. However, these models cannot recapitulate the hierarchical architecture based on 3D myofibers and the surrounding connective tissue in which the abnormal ECM remodeling occurs. Moreover, the random orientation of 2D myofibers does not allow to create different cellular compartments [17] and to quantify contraction forces [18]. To overcome these limitations, biofabrication of 3D human muscle models has emerged as a promising technology to obtain more complex architectures and to mimic the intricate muscle microenvironment by combining multiple cell populations, 3D ECM and biochemical stimuli. These 3D human skeletal muscle models allow the study of the cell-cell interactions that underlie the onset and progression of fibrosis, while also helping to identify potential therapeutic targets and assess the response to novel therapies [19,20]. Several muscle constructs have been recently developed showing an active contraction through the application of chemical, optical, mechanical and electrical stimulation [21], either continuous [22] or intermittent [[23], [24], [25]].

However, the development of complex muscle tissue microenvironments (e.g. integration of ECs and fibroblasts) has been more challenging, particularly for disease modeling application [26] including dystrophies [27]. Previous works have focused on obtaining vascularized networks in 3D co-cultures of muscle cells and ECs [[28], [29], [30]]. Kim and colleagues engineered human skeletal muscle tissues featuring three distinct coaxial layers of ECs, fibroblasts, and skeletal muscle cells [31]. The authors discovered that including fibroblasts in vascularized muscle tissues boosts both contractile force and angiogenesis. In our previous work, we designed a muscle microenvironment promoting EC differentiation towards a muscle-specific phenotype and the spontaneous self-assembly of myofibers, which were enveloped by a continuous endomysium-like structure [32]. Furthermore, we applied the same model for replicating the fibrotic conditions observed in DMD. Nevertheless, these models fall short in reproducing the overall complexity of skeletal muscle microenvironment and in analyzing how specific cell populations (e.g. vascular cells, immune cells) contribute to drive the early events of muscle fibrosis. In particular, no available in vitro model has enabled the study of EC plasticity and EndoMT within human skeletal muscle, despite its potential role as an early driver of fibrogenesis. Hence, the development of more advanced, reproducible and scalable models of skeletal muscle microenvironment better embracing muscle complexity is of high biological and translational relevance. Our work addresses this gap by developing a 3D mesoscale human skeletal muscle model capable of replicating muscle complexity and contraction upon stimulation under physiological and pathological conditions. Moreover, this model closely mimics the native muscle microenvironment by integrating fibroblasts, vascular and immune cells and replicating both fibrotic and healthy conditions. Importantly, our model uniquely enables the study of EndoMT in skeletal muscle, demonstrating for the first time that both pro-fibrotic cells (i.e. DMD fibroblasts) and an inflammatory microenvironment (i.e. M1 macrophages) can initiate EC trans-differentiation, from macroscale vascular architecture down to the molecular level.

## 2 Methods

### 2.1 Microfabrication of the mesoscale muscle-on-a-chip

Custom-built 3D negative molds and inserts were microfabricated by 3D printing using the FREEPRINT® model 2.0 resin (DETAX GmbH, Germany) after being designed and sketched using a computer-aided design (CAD) software. Liquid Polydimethylsiloxane (PDMS, 184 Silicone Elastomer Kit) was obtained after thoroughly mixing the polymer elastomer base and curing agent (10 parts elastomer to one part curing). Subsequently, the uncured PDMS was poured inside the 3D printed negative mold. After curing (2h and 30 min at 65 °C), the PDMS structures were sterilized with isopropanol. The resulting structure was 35x25x5 mm^3^ characterized by a central empty channel (8x2x1.6 mm^3^ where 2x1.6 mm^2^ is the cross-sectional area) at the base of the device flanked by two reservoirs. 3D inserts were microfabricated in order to be tucked in the two reservoirs at the sides of the central channel. The device featured an anchoring system with two cylindrical PDMS pillars, one at each end of the central channel, fabricated from a thin PDMS membrane. This membrane was cast using a laser-cut Polymethylmethacrylate (PMMA) mold and subsequently microfabricated into cylindrical pillars using a biopsy punch. Afterward, the fabricated pillars were inserted in the precise gaps designed to host them within the PDMS device.

### 2.2 Cell culture

Human skeletal muscle myoblasts (HSMMs, Lonza CH) were cultured following the vendor's instructions. Muscle cells were grown in muscle growth medium (GM) composed of Dulbecco's Modified Eagle Medium (DMEM, Gibco) supplemented with 20 % V/V Fetal Bovine Serum (FBS, Hyclone), 10 ng/ml epidermal growth factor (EGF, PeproTech), 5 ng/ml basic fibroblast growth factor (bFGF, PeproTech), 0.4 μg/ml Dexamethasone (Sigma-Aldrich), 1 % penicillin/streptomycin (P/S, Gibco), 1 % L-glutamine (Gibco).

For myoblast fusion and differentiation, cells were switched to differentiation medium (DM) containing low glucose DMEM (Gibco) supplemented with 2 % V/V horse serum (Hyclone), 10 μg/ml Insulin (Sigma-Aldrich), 1 % P/S (Gibco), 1 % L glutamine (Gibco). Experiments were performed on early passages (5th-9th) of HSMMs.

Human dermal microvascular endothelial cells (HDMECs) were purchased from Angioproteomie and cultured following the vendor's instructions. The company certifies that HDMECs are positive for VWF (von Willebrand factor)/Factor VIII and cytoplasmic PECAM1 (Platelet Endothelial Cell Adhesion Molecule-1). Cells are tested for cytoplasmic uptake of Di-I-Ac-LDL (DiI-conjugated acetylated low-density lipoprotein). HDMECs were grown in Endothelial Cell Growth Medium-2 (EGM™-2 MV, Lonza), with flasks previously treated with gelatin-based coating solution (CellBiologics). Starting from the 5th passage, HDMECs were detached, counted, and used for the seeding of the stromal compartment.

Healthy Muscle Fibroblasts were isolated from human tissue biopsies based on previously established protocols [33,34]. Briefly, fibroblasts were isolated from connective tissue chunks of muscle biopsies. Connective tissue was minced into small pieces and plated on standard plastic dishes. Muscle-derived fibroblasts from DMD patients were provided by the Italian Telethon Network of Genetic Biobanks. Fibroblasts were obtained by immunomagnetic selection from primary cell cultures derived from muscle biopsies of three DMD patients (aged 3–6 years). Muscle fibroblasts (healthy and DMD) were cultured using DMEM supplemented with 15 % FBS, 1 % P/S, 1 % L-glutamine and 5 ng/ml bFGF. Experiments were performed on early passages (4th-8th) of healthy and DMD fibroblasts. Cells were detached, counted, and used for the seeding of the stromal compartment.

Human primary monocytes were isolated from healthy donor blood donations within 24 h (approved by Ethical Committee protocol CE-3428 at IRB). Peripheral blood mononuclear cells (PBMCs) were obtained via Ficoll-hypaque density centrifugation, followed by CD14^+^ monocyte isolation using positive immunoselection (130-050-201, CD14 MicroBeads, Miltenyi Biotec). One million CD14^+^ monocytes were cultured in a 24-well plate in macrophage differentiation media comprising RPMI-1640 supplemented with 10 % Fetal Bovine Serum, 1x nonessential amino acids, 1 mM Sodium pyruvate, 20 mM GlutaMAX, 50 μM β-Mercaptoethanol, Penicillin 50 U/ml – Streptomycin 50 μg/ml (all from Gibco, Thermo Fisher Scientific) and 50 ng/ml of recombinant human granulocyte-macrophage colony-stimulating factor GM-CSF (mGMP-rHuGM-CSF, Gentaur Molecular Products). After 5 days, M0 macrophages were polarized to M1 by stimulation with 200 ng/ml Lipopolysaccharide (LPS, Sigma-Aldrich) and Interferon (IFN)-γ (Biolegend). After 24 h, M1 macrophages were detached and used for co-culture.

### 2.3 Biofabrication of muscle constructs

HSMMs (15 × 10^6^ cells/ml) were mixed with a hydrogel formulation consisting of 18.5 % v/v fibrinogen (dissolved in Phosphate-Buffered Saline - PBS, final concentration 4 mg/ml, Sigma-Aldrich), 20 % v/v Matrigel ® Growth Factors-Reduced (Corning), 57.5 % v/v GM and 4 % v/v Thrombin (dissolved in 0.1 % BSA, final concentration 4 U/ml, Sigma-Aldrich) for a total volume of 20 μL. HSMMs were resuspended in GM, mixed with the hydrogel, and the resulting cell-hydrogel mix was pipetted directly inside the empty channel of the device containing Pluronic F-127 (Sigma-Aldrich) pre-coated inserts to reduce cell adhesion.

The cell/hydrogel mixture was left to polymerize at 37 °C for 30 min before adding GM supplemented with 1.5 mg/mL 6 aminocaproic acid (ACA, Sigma-Aldrich). The next day, differentiation was induced by switching to DM with 2 mg/mL ACA. The DM was changed every other day.

### 2.4 Biofabrication of the stromal compartment: one-step seeding

The first study compared healthy and fibrotic muscle models. Such models were based on the direct integration of healthy or DMD muscle fibroblasts in the myobundle. Cells were combined in order to have three different conditions: HSMM (HSMM-only), F. DMD (HSMMs and Duchenne fibroblasts) and F. DMD + N (HSMMs and Duchenne fibroblasts treated with Nintedanib). Differences among conditions were based on cell sources and cell ratio. HSMM-only condition is the myobundle obtained from primary myoblasts, while DMD conditions had a HSMM:fibroblast ratio of 10:1. Models were cultured for a week with muscle differentiation medium. On day 7, the models were exposed to electrical stimulation. From day 8, force quantification was performed.

### 2.5 Biofabrication of the stromal compartment: two-steps seeding

In the second study, healthy, fibrotic and inflammation models were generated. The models were based on a myobundle of HSMMs surrounded by a second hydrogel containing a mixture of HDMECs, healthy or DMD fibroblasts, and/or M1 macrophages. Fibroblasts and HDMECs were combined in order to have two different conditions, namely Healthy control (Healthy fibroblasts and HDMECs) and Fibrosis (DMD fibroblasts and HDMECs). An additional condition, Inflammation, was designed to mimic an inflammatory environment by co-culturing healthy fibroblasts, HDMECs, and M1 macrophages. To obtain this arrangement, a two-steps seeding was required. In the first one, the HSMMs were seeded to generate the myobundle and then grown in muscle GM for 1 day followed by differentiation with muscle DM for a week. In the second step, stromal cells were added to the surrounding hydrogel, tailored to the specific conditions described above. EGM™-2 MV (deprived of Hydrocortisone) was used as the growth medium for the co-culture experiments.

For each cell solution, the density of HDMECs and fibroblasts (Healthy or DMD) was 3 × 10^6^ cells/ml and 6 × 10^5^ cells/ml, respectively. The M1 density was 6 × 10^5^ cells/ml. In contrast with the myoblast seeding (see paragraph 2.3), here the gelling solution consisted only of fibrinogen (5 mg/ml) and EGM™-2 MV medium. The cell/hydrogel mixture was pipetted onto the myobundle and allowed to polymerize for 30 min at 37 °C followed by incubation in EGM™-2 MV containing 2 mg/ml ACA. Tissues were maintained at 37 °C with 5 % CO_2_. The media was changed every other day.

### 2.6 Cryopreservation and cryosectioning

Constructs were fixed with 2 % paraformaldehyde for 16 ± 2 h. Fixed myobundles were treated with sucrose (Sigma-Aldrich) solutions (in PBS) for cryopreservation: 15 % sucrose in PBS until tissue sinks (6–12 h) and then 30 % sucrose in PBS for 16 ± 2 h. For snap-freezing, 2-methyl butane (Sigma-Aldrich) was first chilled on dry ice. Meanwhile, the myobundles were embedded in OCT (Optimal cutting temperature) embedding matrix (CellPath). After reaching 2-methyl butane's optimal freezing temperature, the molds containing the OCT-embedded myobundles were placed on top of it and let freeze completely. Samples were then stored at −80 °C until their use. Myobundle cryosections were cut at 50 μm increments using the CryoStar NX50 (Thermo-scientific) cryostat and put on SuperFrost™ microscope slides (Thermo-Scientific). These slides were then stored at −20 °C.

### 2.7 Paraffin embedding and tissue sectioning

Fixed myobundles were soaked in 70 % ethanol for 16 ± 2 h before the paraffin embedding. The following day, the myobundles were subjected to the paraffin processing schedule (Table S1, supplementary material), and the final paraffin embedding using STP120 tissue processor and Myr EC 350 (histocom AG, CH) respectively. The paraffin-embedded constructs were then stored at room temperature until further use. Tissue blocks were sectioned both transversely and longitudinally using a standard rotary microtome (Leica, Germany). The collected sections were either 8 μm or 20 μm thick. These sections underwent a deparaffinization process (Table S2, supplementary material) to remove the wax, followed by either H&E staining or immunofluorescence staining. The stained sections were mounted and examined microscopically using an inverted microscope (Nikon, NI-E) or confocal microscope (Leica, Germany).

### 2.8 Immunofluorescence

Permeabilization and blocking steps on fixed samples were performed with 0.15 % (for sections) or 0.3 % (for myobundle) Triton X-100 (Sigma-Aldrich) and Bovine Serum Albumin (5 %) respectively. Samples were then probed with the following primary antibodies (for 16 ± 2 h at 4 °C):anti-α Smooth Muscle Actin (1:200, mouse anti-human, Abcam); anti-Fast Myosin Heavy Chain (1:200, mouse anti-human, Abcam), anti-Sarcomeric Alpha Actinin (1:200, mouse anti-human, Sigma-Aldrich); anti-Dystrophin (1:200, rabbit anti-human, Abcam); anti-FSP1 (1:200, rabbit anti-human, Sigma-Aldrich); anti-Collagen type I (1:250, rabbit anti-human, Abcam). Moreover, Filamentous Actin (F-actin) staining was performed with Phalloidin-iFluor 647 (1:1000, Abcam). Afterward, samples were washed with PBS and incubated for 3–4 h with secondary antibodies at 4 °C. Fluorescent-labeled goat (anti-rabbit or anti-mouse) Alexa Fluor 488, Alexa Fluor 568, Alexa Fluor 633 or Alexa Fluor 647 by Invitrogen were used. Nuclear staining was performed with NucBlue™ Fixed Cell ReadyProbes™ Reagent (DAPI, Invitrogen) by adding a few drops onto the samples. Vybrant cell labeling (DiD, LifeTech) was employed to track muscle-derived fibroblasts (Table S3, supplementary material). Samples were kept in PBS at 4 °C before imaging.

Fluorescent images were taken with BioTek Lionheart FX Automated Microscope (Agilent Technologies), Inverted Fluorescence Microscope Eclipse DS-Fi2 (Nikon) or STELLARIS 8 confocal microscope (Leica Microsystems). Details are reported in each figure.

### 2.9 Image analysis

Stained samples were imaged using either the Leica DMi1 inverted microscope (Leica, Germany), with the BioTek Lionheart FX Automated Microscope (Agilent Technologies) or the STELLARIS 8 confocal microscope (Leica, Germany) equipped with LAS software (Leica, Germany), using 10 × and 20 × magnifications. Images were then analyzed using ImageJ version 1.52i. The software was used for detecting myobundle diameter, percentage of nuclei in each myofiber (i.e. fusion index), myofiber directionality and pillar displacement. Details are reported in each figure.

### 2.10 Collagen quantification

To quantify the secreted collagen (Types I-V) in the different models, a colorimetric Collagen Assay Kit (Sigma-Aldrich) was used. After 24 h stimulation, constructs were snap-freezed in liquid nitrogen and eventually stored at −80 °C. For the analysis, frozen models were resuspended in Acetic Acid and 1 s-long sonicator pulses were used for tissue homogenization. After centrifugation, the supernatant was used for the analysis. The quantification was performed following the manufacturer instructions. Briefly, the pH was balanced with NaOH solution and an enzymatic digestion of collagen was performed, exposing N-terminal glycine containing peptides that reacted with a fluorescent dye. The fluorescent intensity, directly proportional to the collagen concentration in the sample, was measured with a fluorescence plate reader (Tecan) and normalized against standard concentrations.

### 2.11 Electrical stimulation

In order to perform electrical stimulation from D7, custom-made platforms were used hosting six devices each, hence allowing the stimulation of six myobundles in parallel. The base of the platform was 3D printed by Fused Deposition Modeling (FDM) technology using the SideWinder-X2 3D Printer (Artillery) and PolyLite PLA (Polymaker) material. For the platform lid, a 5 mm thick PMMA (polymethyl methacrylate) sheet was cut using a laser cutting machine. The lid design features twelve rectangular small holes to accommodate the electrodes. The electrodes were made of carbon silicone and were arranged in pairs, with two electrodes for each myobundle. The platforms were washed in distilled sterile water and sterilized with 70 % isopropanol and UV light before use.

The electrical stimulation system used was a PC-based system. The system included a Data Acquisition (DAQ) device (National Instruments) directly connected to the PC, a dual power supply ( ± 40 V), and an amplification system (consisting of an amplifier printed circuit board (PCB), an operational amplifier (OP-AMP) and a power transistor) (see Fig S4, supplementary material). The DAQ control board had six output channels that could lead to potentially stimulating six platforms (therefore 36 devices) in parallel with different stimulation regimes. Electrical stimulation protocols were written in a graphical programming software. Muscle myobundles were stimulated using bipolar electrical impulses with an electric field density between 1 V/mm and 13 V/mm. Specifically, myobundles were continuously electrically stimulated at 1 Hz for 1 s every 2 s.

### 2.12 Finite element method (FEM) simulations: pillar displacement

To estimate the elastic properties of PDMS pillars, the Comsol software was used (COMSOL Multiphysics, Stockholm, Sweden). The geometry was imported by a CAD model made in Solidworks software. A fixed constraint was set on the basal face of the pillar, while a boundary load was applied in a range of different forces and locations (further details below). The PDMS properties were already defined in the Comsol material library. Tetrahedral mesh was generated dividing the simulated volumes into up to 25 × 10^3^ elements. A parametric study was performed defining different applied forces (μN) and different application points (μm). The forces considered were 5, 25, 50, 125, 250, 500, 750 and 1000 μN. The application points (height from the basis of the pillar) ranged from 500 to 1700 μm with steps of 100 μm (Table S4, supplementary material).

### 2.13 Mechanical profiling of the pillars

To validate the FEM simulations, experimental quantifications of the Force-Displacement properties were performed on PDMS pillars. Two parameters were tested: pillar diameter (0.5 mm and 0.75 mm) and PDMS elastomer-to-curing agent ratios (10:0.5 and 10:1). To perform the analysis, FT-S Microforce Sensing Probe (Femtotools AG) was used. The sensing tip was moved against the fixed anchor for a range of 200 μm with 5 μm steps (see Videos S1-S4, supplementary material), for each specific application point. A log file with the forces measured for all the positions was generated. The measurement was taken on three replicates per condition. From these measurements, the elastic constant of the PDMS pillars was derived as the slope of the force – deformation curve, providing the experimental input required to benchmark the FEM predictions.

### 2.14 Contraction force assessment through displacement measurements

The mechanical profiling of the pillar generated a data file containing values for height, displacement, and force. These values were interpolated using MATLAB, and a second-order polynomial model was applied to construct a surface plot and refine the interpolation. A short-frame video capturing myobundle contraction (see Video S5, Supplementary Material) was analyzed to quantify contraction force. This analysis enabled precise measurement of visible pillar displacement and the attachment height of the myobundle to the pillar. These precise values were then given as inputs to the model, allowing a non-invasive optical quantification of contraction force.

### 2.15 FEM simulation: electric field intensity

To study the Electric field distribution in the device, the Comsol software was used. A cross-section of the geometry (1 mm thick) was imported from the whole CAD model made in Solidworks software. For the fibrin hydrogel, the relative permittivity was defined as 80.1 and electrical conductivity was set at 1.5 S/m. Tetrahedral mesh was generated dividing the simulated volumes into up to 2.8 × 10^5^ elements. The distribution of the electric field (V/mm) was quantified for the horizontal and vertical diameters of the myobundle.

### 2.16 Sample digestion, staining & flow cytometry

To recover HDMEC from 3D Matrigel/fibrin hydrogels, a combination of Nattokinase (NSK-SD, JBSL-USA) and Collagenase-I (Worthington-Biochemical) was utilized. The hydrogels were treated with a solution containing 500 U/ml Nattokinase and 5 mg/ml Collagenase-I in PBS. After 15 min of digestion at 37 °C on a thermoshaker (Biosan), the solution was filtered through 100 μm cell strainers (ClearLine®), and digestion was blocked. The cells were then transferred to Fluorescence-activated cell sorting (FACS) tubes, centrifuged, and resuspended in PBS + 1 % Draq7 (abcam) to assess cell death. After incubation and washing with MACS buffer (PBS + 10 % FBS + 2 mM EDTA), cells were stained with fluorochrome-conjugated antibodies (Table S5, supplementary material), washed again, and fixed in 1 % paraformaldehyde in PBS for analysis on an LSR Fortessa™ Cell Analyzer (BD Bioscience). Data analysis was performed using FlowJo version 10.07.2. Cells were segregated into two groups based on their GFP fluorescence: GFP-positive (GFP+) or GFP-negative (GFP-). Only the GFP + cells (HDMEC) were subsequently analyzed to measure the expression levels of N-Cadherin and CD31/platelet-endothelial cell adhesion molecule-1 (CD31/PECAM-1). Finally, the sorted cells were placed in RNAse-free Eppendorf tubes with Trizol LS and stored at −80 °C until use.

### 2.17 RNA extraction, retrotranscription and qRT-PCR

mRNA extraction from HDMECs was performed using the Direct-zol RNA Miniprep Kit (R2050, Zymo) following the vendor's protocol. Reverse transcription into cDNA was performed in presence of random primers (Invitrogen), deoxynucleotides solution (New England BioLabs) and M-MLV Reverse Transcriptase (Invitrogen). Gene expression of selected markers was analyzed by qRT-PCR. In particular, quantitative analysis of PECAM1 (CD31, Hs01065279_m1), CDH2 (N-cadherin, Hs00983056_m1), KDR (Hs00911700_m1), PAI1 (Hs00167155_m1) and SNAI1 (Hs00195591_m1) gene expression was performed using TaqMan™ gene expression assay probes (Applied Biosystems™). The gene expression was normalized to the housekeeping gene ribosomal protein 18S (Hs99999901_s1) using the 2-ΔΔCt method.

### 2.18 RNA extraction, DNase treatment and RNA sequencing

First, sample preparation began with gel digestion of muscle constructs using Trizol (TR118 MRC), followed by phase separation with 1-Bromo-3-chloropropane (B9673). RNA extraction from muscle cells was then performed according to *ReliaPrep*™ *RNA Miniprep* Systems’ protocol (Promega), which included DNase treatment. The purified RNA was subsequently subjected to bulk RNA sequencing and analysis. NEBNext UltraExpress RNA Library Prep Kit for Illumina (New England BioLabs Inc.) was employed with the NEBNext Multiplex Oligos for Illumina (New England BioLabs Inc.) and NEBNext® Poly(A) mRNA Magnetic Isolation Module for cDNA synthesis with the addition of barcode sequences. The pre-pool sequencing was performed using the NextSeq2000 (Illumina, San Diego, CA, USA) with the P2 reagents kit V3 (100cycles; Illumina). Samples were processed starting from stranded, single-ended 120bp-long sequencing reads.

We evaluated the RNA-seq reads quality with FastQC (v0.11.5) and removed low-quality reads/bases and adaptor sequences using Trimmomatic (v0.35). The trimmed-high-quality sequencing reads were aligned using STAR, a spliced read aligner allowing sequencing reads to span multiple exons. On average, we aligned 87 % of the sequencing reads for each sample to the reference genome (HG38). The HTSeq-count software package was then used to quantify gene expression with GENCODE v22 as gene annotation. Expression values are provided in a tab-delimited format. We subsetted the data to genes with a counts-per-million value greater than 5 in at least one sample. The data were normalized using the ‘TMM’ method from the edgeR package and transformed to log2 counts-per-million using the edgeR function ‘cpm’. The differential gene expression (DEGs) of each comparison of interest was computed using limma based on TMM-normalization and voom transformation. Heatmaps of significant DEGs in any comparison were visualized with pheatmap v1.0.12. We then performed the Gene-set enrichment analysis (GSEA) for each comparison, ranking the genes according to -log10(p-value)∗logFC to identify the enriched biological pathways or gene ontology terms associated with them. Selected gene set enrichment analysis of gene ontology (GO) Kyoto Encyclopedia of Genes and Genomes (KEGG) pathway, and Hallmarks of Cancer from MSigDB was performed with ClusterProfiler v3.8.1. All analyses were conducted in R, and scripts are available upon request to ensure transparency and reproducibility.

### 2.19 Liquid chromatography – tandem mass spectrometry (LC-MS/MS)

Sample preparation began with mincing both muscle myobundles and co-cultured muscle-fibro bundles. The tissues were then snap-frozen and stored at −80 °C until further use. Before mass spectrometry analysis, the samples were treated as biological tissues and underwent lysis in 4 % SDS in 100 mM Tris pH 7.6 with 10 mM DTT by sonication with a Branson Ultrasonics SFX150 cell disruptor (3 cycles of 5 s, 80 % power), followed by sonication in a Bioruptor (Diagenode, 15 cycles, 30s on, 30s off, high mode), and a 10 min incubation at 95 °C in agitation. The lysates were then alkylated with 50 mM iodoacetamide for 30 min at room temperature, cleared by centrifugation, and proteins were precipitated overnight in 80 % acetone at −20 °C. The protein pellets were dried and resuspended in 8M urea in 50 mM ammonium bicarbonate (ABC) by Bioruptor sonication. Digestion was performed with LysC (Wako Fujifilm, 1:100 w/w) for 2 h at room temperature, after which the digestion buffer was diluted to 2M urea with 50 mM ABC and digestion was continued overnight at room temperature with trypsin (Promega, 1:100 w/w). Digestion was stopped in 2 % acetonitrile (ACN) and 0.3 % trifluoroacetic acid (TFA) and the samples were cleared by centrifugation for 5 min at maximum speed. The resulting peptides were purified into C18 StageTips (Rappsilber et al., 2007), and eluted with 80 % ACN, 0.5 % acetic acid. Finally, the elution buffer was evaporated by vacuum centrifugation and purified peptides were resolved in 2 % ACN, 0.5 % acetic acid, 0.1 % TFA for single-shot LC-MS/MS measurements.

Peptides were separated on a nanoElute2 HPLC system (Bruker) coupled via a nanoelectrospray source (Captive spray source, Bruker) to a timsTOF HT mass spectrometer (Bruker). 500 ng of peptides per sample were loaded in water/0.1 % formic acid into a 75 μm inner diameter, 25 cm long column in-house packed with ReproSil-Pur C18-AQ 1.9 μm resin (Dr. Maisch HPLC GmbH), kept at 50 °C in a column oven, and eluted over a 60-min linear 2–35 % gradient of ACN/0.1 % formic acid at a 300 nl/min flow rate. The mass spectrometer was operated in a data-independent (DIA)-PASEF mode with 100 ms of accumulation and ramp time, covering with 21 mass steps, 25 Da wide, and 1 mobility window, a mass range from 475 to 1000 Da and a mobility range from 0.85 to 1.27 Vs cm-2, with an estimated cycle time of 0.95 s.

DIA raw data were searched with DIA-NN version 1.8.1 (Demichev et al., 2020) with default settings, against a deep learning-based predicted library generated from the human Uniprot database (February 2024). For library generation, FASTA sequences were in-silico digested with Trypsin/P with 1 missed cleavage, enabling N-terminal methionine excision and cysteine carbamidomethylation as a fixed modification. Precursor and fragment mass tolerances were determined automatically for each run separately and ranged from 10 to 20 ppm. Proteomic analysis was performed using raw data, generating two separate heatmaps. The first heatmap was created by analyzing proteins consistently detected across all conditions and experiments, allowing for a comparative assessment of three conditions: HSMM, F.DMD, and F.DMD + N. Protein expression levels were averaged (among experiments) per condition and further normalized to the control HSMM condition for relative comparison. The second heatmap focused specifically on the two fibrotic conditions (F.DMD and F.DMD + N), comparing protein expression levels between the Nintedanib-treated and untreated conditions. Results are presented as the ratio of protein expression in F.DMD + N relative to F.DMD (F.DMD + N/F.DMD).

### 2.20 Statistical analysis

Statistical analysis was performed using GraphPad Prism (v.8.0) software. Data were first analyzed for normality and then compared with parametric statistics. Unpaired student's t-test or one-way ANOVA were used to assess statistical significance. Differences were considered significant with p < 0.05 (∗), p < 0.01(∗∗), p < 0.001(∗∗∗) and p < 0.0001(∗∗∗∗). Results are presented as mean ± standard deviation (SD). Details are reported in each figure.

## 3 Results

### 3.1 Design and biofabrication of the mesoscale muscle-on-a-chip

**Fig. 1:**
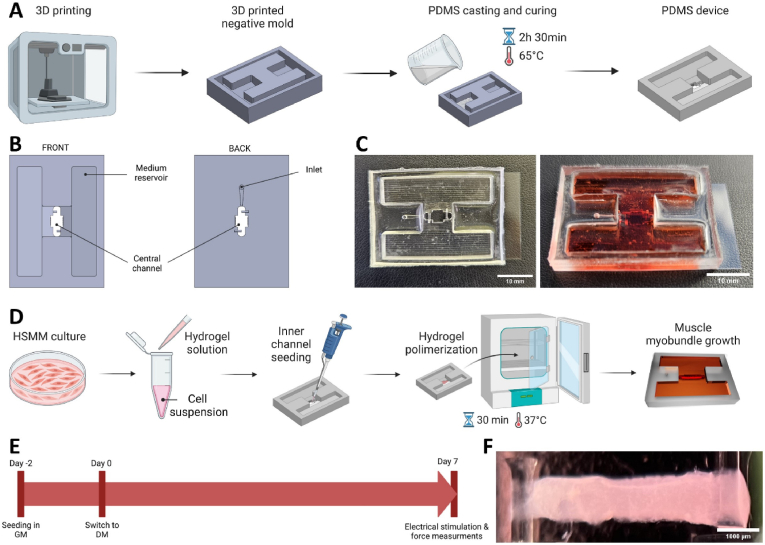
Design and development of the muscle-on-a-chip. (A) Schematic of device microfabrication. (B) CAD design of the front and back views of the mesoscale device. (C) PDMS device bonded to a glass coverslip. Scale bar = 10 mm. (D) Human primary skeletal muscle myoblast (HSMM) seeding workflow. (E) Schematic timeline for the generation of 3D myobundles from HSMMs. GM: growth medium. DM: differentiation medium. (F) Detail of the myobundle within the PDMS device. Scale bar = 1000 μm.

Mesoscale devices hosting muscle myobundles were microfabricated through a multi-step process. The workflow for obtaining a poly-dimethyl-siloxane (PDMS) chip via the replica molding technique is illustrated in Fig. 1A. The mesoscale chip includes a 2 mm thick PDMS chamber with a central channel and two reservoirs, flanked by two parallel PDMS pillars on each side. It has a 1 mm diameter inlet and a medium reservoir (Fig. 1B and Fig. S1, supplementary material). The bottom is bonded to a 500 μm thick coverglass using plasma bonding (Fig. 1C). The muscle tissue is formed biofabricated within the central channel and attaches to the two pillars.

Engineered human skeletal muscle bundles were obtained using primary HSMMs suspended in a hydrogel mix (Fig. 1D), based on previously published works [22,35]. The cell-hydrogel suspension was pipetted into the central channel surrounding the PDMS pillars. After polymerization of the hydrogel mixture, medium was added, and culture was initiated. Following a two-day hydrogel compaction period in growth media, low serum differentiation media was used to induce myobundle formation and differentiation (Fig. 1E). The myobundles were allowed to differentiate for one week (Fig. S2, supplementary material) then electrical stimulation was applied, and contraction force was measured. Myobundles were kept in culture for up to 30 days after seeding. During this culture time, the PDMS pillars within the channel acted as tendon-like anchors, establishing uniaxial tension during myobundle remodeling and directing tissue formation and compaction (Fig. 1F).

### 3.2 Optimization of the pillar mechanical properties

Although pillar-based setups have been employed in previous human 3D muscle models [[36], [37], [38], [39]], the unique dimensions of our device required an initial characterization of various pillar designs to determine the most suitable option for our model.

**Fig. 2:**
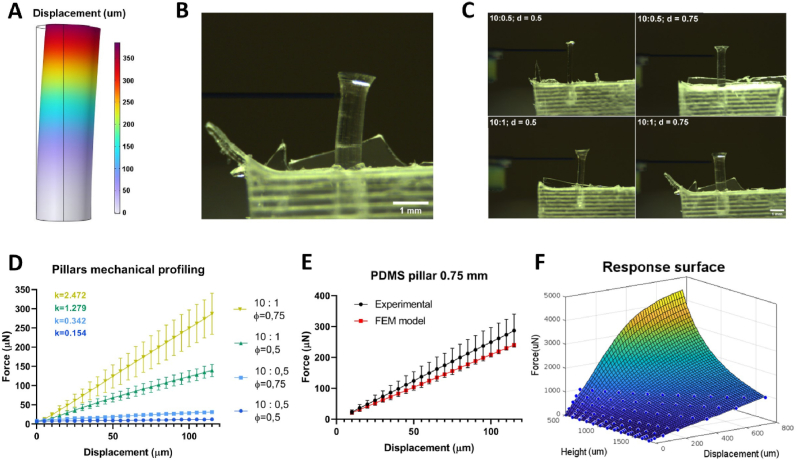
Mechanical characterization of the pillar. (A) Finite element method (FEM) simulation of the PDMS pillar displacement (pillar diameter 0.75 mm). B) Representative image of pillar displacement using nanoindentation: detail of the probe in contact with the pillar. C) Mechanical profiling of the PDMS pillars in four combinations of diameter (0.5 and 0.75 mm) and PDMS base:curing agent ratio (10:0.5 and 10:1). Scalebar = 1 mm. D) Plots of the measured Force-Displacement ratio and extraction of the spring constant. N = 3. E) FEM predicted Force-Displacement ratio (red) confirmed by the experimental mechanical profiling (black line) of the PDMS pillar with 0.75 mm. N = 3. F) Response surface of the 0.75 mm (diameter)-10:1 (base:curing agent ratio) pillar obtained by interpolating indentation height, pillar displacement and force. (For interpretation of the references to color in this figure legend, the reader is referred to the Web version of this article.)

Two variables were evaluated to optimize the elastic properties of the PDMS pillars: the pillar diameter and the ratio between the PDMS elastomer base and the curing agent. Diameters of 0.5 mm and 0.75 mm were considered. The PDMS component ratios evaluated were 10:0.5 and 10:1, with the 10:1 ratio achieving a higher degree of polymerization. A finite element model (FEM) of the PDMS pillar was developed (Fig. 2A) to compute pillar displacement based on two variables: the force generated by the myobundle contraction and the attachment point of the myobundle on the pillar (Table S4, supplementary material). It was found that displacement increased as the myobundle attachment point moved farther from the base of the pillar, while the generated force remained constant. Mechanical profiling using a micro-force indenting sensor (Fig. 2B-C) quantified the force-displacement relationship (Fig. 2D), revealing major differences in rigidity between the conditions (see Videos S1-S4, supplementary material). Pillars with a 10:0.5 PDMS ratio exhibited extremely high flexibility compared to the 10:1 ratio. Specifically, for PDMS with a 10:1 ratio the values were 2.472 (⌀ = 0.75 mm) and 1.279 (⌀ = 0.5 mm), while for PDMS with a 10:0.5 ratio the constants dropped to 0.342 (⌀ = 0.75 mm) and 0.154 (⌀ = 0.5 mm). Therefore, the 10:1 PDMS pillars with 0.75 mm diameter were selected for further analyses having an elasticity more compatible with the expected force generated by the myobundles. The computational model was validated by comparing the force-displacement ratio obtained in the simulations with the mechanical profiling of the selected pillar (Fig. 2E). The results were used to create a response surface (Fig. 2F), from which a polynomial function was derived (Fig. S3, supplementary material). Importantly, this function enabled the calculation of the force generated by the myobundle based on the observed pillar displacement and attachment point height, a parameter that is neglected in current 3D muscle models, hence affecting the comparison of literature results

### 3.3 Design of the electrical stimulation system

**Fig. 3:**
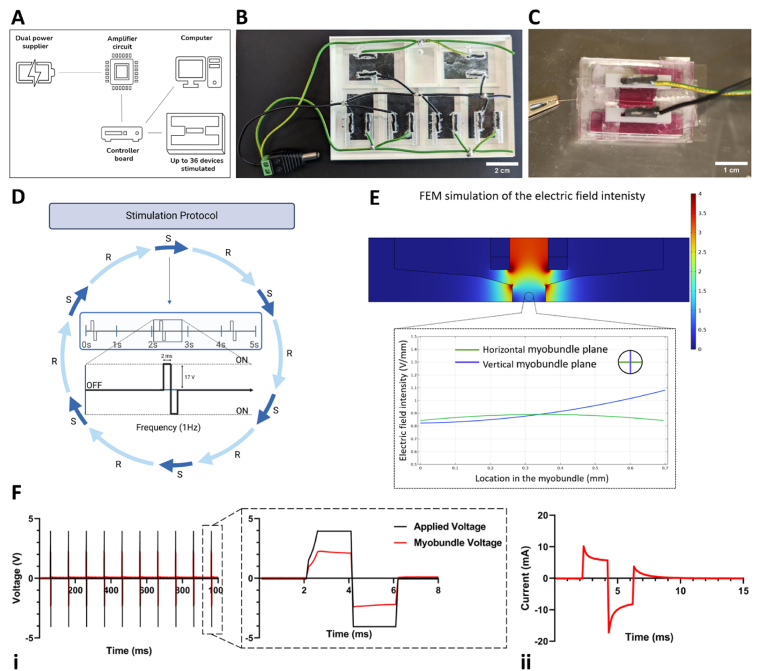
Electrical stimulation setup. A) Graphical representation of the custom-made electrical stimulation system. B) Electrical stimulation platform with base (white), PMMA cover and electrodes inserted in the top lid and connected to the wires. Scalebar = 2 cm. C) Representative image of the mesoscale device being stimulated. Scalebar = 1 cm. D) 24h stimulation cycle consisting of 1 h Stimulation (S) and 3 h Rest (R) periods. E) Cross-section view of the FEM computational simulation of the Electric Field distribution in the model. F) Experimental testing of the electrical stimulation system i) Voltage applied over time; ii) Electric current generated over time.

To mimic the function of skeletal muscle tissue in vitro, electrical pulses were delivered to the muscle construct. A custom-made electrical system was developed to guarantee maximum flexibility during the optimization of the stimulation program (Fig. 3A and Fig. S4, supplementary material). Remarkably, this system allowed to stimulate up to 36 independent devices with two different stimulation regimes. Custom-designed platforms were used, consisting of a base housing the devices and a cover where the electrodes were attached (Fig. 3B and Fig. S5, supplementary material). The electrodes were placed in the central channel, parallel to the myobundle, and aligned with the devices to ensure immersion in the culture medium (Fig. 3C). To reduce the negative chemical effects of the electric field on the distribution of charges in the culture medium, a squared bipolar pulse stimulation pattern was used (Fig. 3D). A FEM computational simulation was performed to quantify the distribution of the electric field in the model (Fig. 3E). The simulation results predicted a 1 V/mm electric field intensity on the myobundle. An experimental verification of the system was then performed (Fig. 3F). A 10-Ω resistor was connected in series with the device, and the voltage drop was measured using the DAQ I/O controller board (Fig. 3F–i). According to Ohm's law, the current in the channel was estimated by dividing the measured voltage by the 10-Ω resistance. This current was 10 mA when 4 V was applied (Fig. 3F–ii). Additionally, an electrode placed at the location of the myobundle directly measured the voltage, which was half of the input voltage, thus confirming the system functionality.

### 3.4 Characterization of the human myobundles

**Fig. 4:**
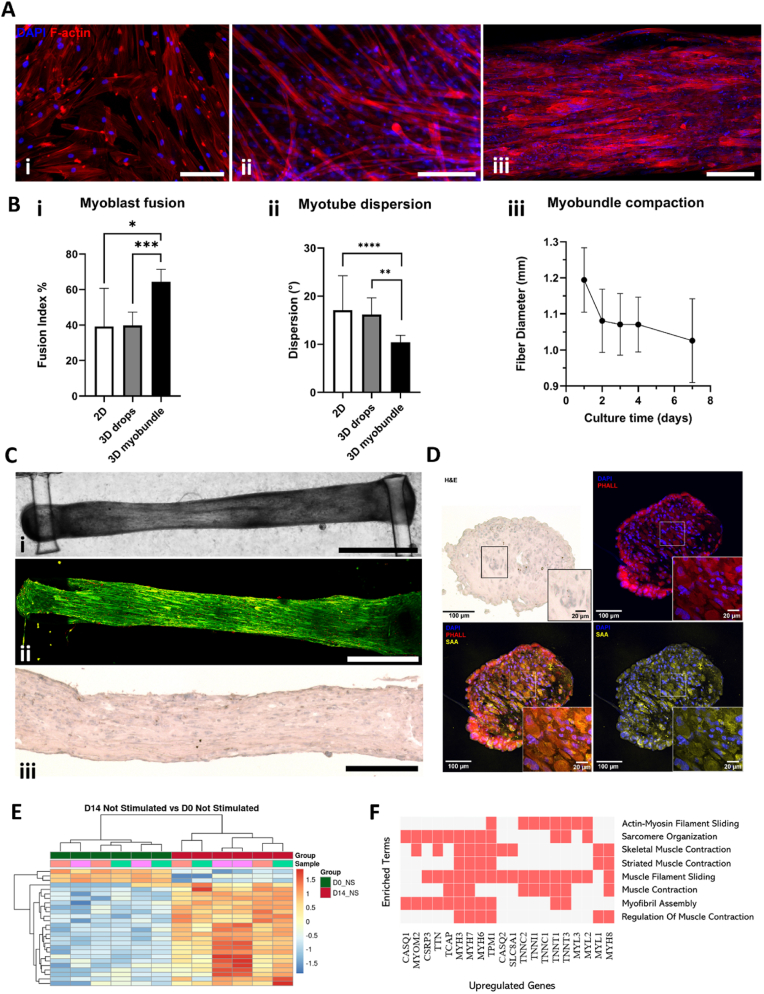
Biofabrication and characterization of the muscle compartment. (A) Different seeding conditions (2D, 3D drops and 3D myobundle. Scale bar: 100 μm. (B) Fusion index (i) and Dispersion rate (ii) comparison between different culture supports: 2D flat, 3D drops, and 3D myobundle (N = 5). Unpaired *t*-test analysis: ∗p < 0.05 ∗∗p < 0.01, ∗∗∗p < 0.001, ∗∗∗∗p < 0.00001; iii) myobundle diameter over 8 days of differentiation. (C) Representative images of the human skeletal muscle bundle after 7 days of differentiation: i) brightfield picture, scale bar = 2 mm; ii) Live&Dead assay, scale bar = 1 mm; iii) H&E stained longitudinal myobundle section, scalebar = 200 μm. (D) Transversal cryosections of the myobundle: immunofluorescent images of actin (phalloidin) and Sarcomeric Alpha Actinin (SAA); nuclei counterstained with DAPI. (E) RNAseq of the myobundles at day 14 (without electrical stimulation) vs. day 0 of differentiation. ∗p < 0.1, N = 3. F) Biological processes (and associated genes) upregulated at day 14 vs. day 0 of differentiation.

The myobundle setup, in which the construct is suspended between two pillars, induced passive unidirectional stretching, effectively promoting muscle cell differentiation and alignment, an outcome not achievable with conventional 2D cultures or 3D bioprinting approaches. To validate this setup, three different seeding conditions were compared: 2D flat culture dishes, 3D fibrin/matrigel drops, and 3D myobundles. The 3D myobundle setup exposed the differentiating muscle cells to passive uniaxial strain, significantly enhancing myotube alignment and myoblast fusion compared to the other conditions (Fig. 4A and B and Fig. S6, supplementary material).

After one week of culture, immunofluorescence analysis targeting F-actin, a highly expressed cytoskeletal protein, was performed for each condition (Fig. 4A). Images were processed to quantify the fusion index (see Fig. 4B–i). This index indicates the extent of myoblast fusion into myotubes, which was measured as 39.37 ± 12.9 % for the 2D flat setup, 45.1 ± 13.1 % for the 3D drops, and 60.7 ± 7 % for the myobundle. These findings identified the myobundle as the optimal configuration for promoting muscle differentiation. Additionally, Fourier spectrum analysis was used to examine the structural orientation of the myotubes. Histograms generated with Fiji software illustrated the distribution of orientation angles (degrees from the horizontal axis) for each condition (see Fig. S6, supplementary material). The myobundle condition exhibited a directionality distribution closely resembling a Gaussian distribution. To statistically analyze these differences, the dispersion (representing the standard deviation of the Gaussian distribution) was compared among the three groups. The average dispersion for the 2D flat dishes and 3D drops was similar, measuring 17.1 ± 7.1 and 16.2 ± 3.4°, respectively. In contrast, the 3D myobundle average dispersion was 10.4 ± 1.46°, demonstrating its major ability to promote myotube alignment (see Fig. 4B–ii).

During the first week of differentiation, progressive myofiber alignment was evidenced by a reduction in tissue diameter, from approximately 1.23 ± 0.07 mm on day 1–1.03 ± 0.04 mm on day 7 (Fig. 4B–iii). Brightfield (Fig. 4C–i) and live/dead (Fig. 4C–ii) imaging confirmed the alignment of individual myofibers, which remained viable throughout the culture period. Furthermore, Hematoxylin and Eosin (H&E) staining of both longitudinal (Fig. 4C–iii) and cross-sectional views (Fig. 4D) as well as the immunofluorescence staining for F-actin and Sarcomeric-Alpha Actinin (SAA), demonstrated that the biofabrication approach rapidly generated dense and compact muscle tissues. Additionally, the average diameter of the myofibers was quantified from the cross-sections as 17.26 ± 2.32 μm (N = 3).

**Fig. 5:**
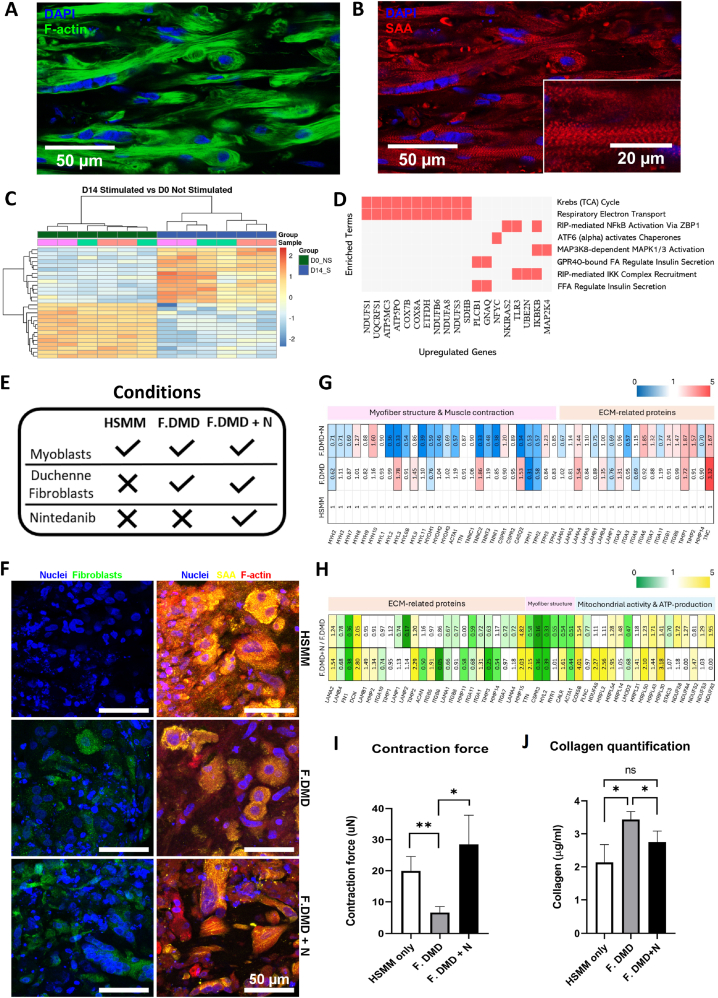
A-B) Longitudinal sections of the myobundle: muscle bundle differentiation evaluated by immunostaining for actin filaments (A) and SAA (B). C) RNAseq of stimulated myobundles at day 14 vs. day 0 of differentiation. ∗p < 0.1, N = 3. D) Enrichment analysis showing upregulated biological processes in stimulated (day 14) vs. non-stimulated (day 14) myobundles. E) Schematic of tested co-cultured conditions for contractility experiments. F) Representative immunofluorescence images of muscle-fibro co-cultures showing nuclei (blue), F-actin (red), SAA (yellow), and Fibroblast-Specific Protein 1 (FSP1, green). HSMM: myoblasts only; F.DMD and F.DMD + N: myoblast-to-fibroblast ratio 10:1. G) Heatmap displaying upregulated and downregulated proteins from mass spectrometry analysis across three conditions (HSMM, F.DMD, F.DMD + N). Results are normalized to only-muscle myobundle (HSMM) protein content (N = 2). H) Heatmap showing upregulated and downregulated proteins in the F.DMD + N condition relative to F.DMD, based on mass spectrometry analysis (N = 2). I) Contraction force quantification of only-muscle, and fibrotic muscle-fibro myobundles with and without Nintedanib. L) Collagen quantification in only-muscle and fibrotic muscle-fibro myobundles with and without Nintedanib. Unpaired *t*-test performed to quantify statistical differences. ∗p < 0.05,∗∗p < 0.01, N = 3. (For interpretation of the references to color in this figure legend, the reader is referred to the Web version of this article.)

To better characterize the maturation level of the myobundles, we conducted a bulk RNA sequencing study. We compared gene expressions in non-stimulated samples from day 14 of differentiation with those from day 0 (Fig. 4E). This analysis revealed 1145 significantly upregulated genes on day 14 compared to day 0. Among these, it is important to highlight key muscle differentiation genes including MYH3, MYH7, TNNT1 and TNNC1. Importantly, further investigation revealed that many of the differentially expressed genes were involved in fundamental biological processes underlying muscle tissue maturation, including sarcomere organization, myofibril assembly, muscle contraction, and calcium handling (Fig. 4F). To specifically address whether the contractile gene isoforms corresponded to developmental or mature states, we analyzed the expression of fetal versus adult isoforms of myosins, troponins, tropomyosins, and myosin light chains. This analysis showed that the majority of isoforms expressed within our myobundles correspond to adult isoforms, which were significantly upregulated on Day 14 compared with Day 0, while fetal isoforms were either reduced or remained low (Fig. S7, Supplementary Material). Overall, these results clearly demonstrate a high level of maturation of the biofabricated myobundles. To further characterize the myobundle, immunofluorescence staining for F-actin and SAA was performed on longitudinal cross-sections (Fig. 5A and B). Immunofluorescence images demonstrated a high level of differentiation in the myobundle through the expression of SAA, which localizes on the Z-discs of the sarcomeres, giving a characteristic striated banding pattern (Fig. 5B).

### 3.5 Electrical stimulation promotes tissue maturation

We next sought to determine the effect of electrical stimulation on muscle tissue maturation. Electrical stimulation was applied from day 7 to day 14, during which we observed systemic, frequency-dependent tissue contractions (see Video S5, supplementary material), evidenced by the displacement of both pillars. On day 14 of differentiation, we performed bulk RNA sequencing on stimulated and unstimulated samples (Fig. 5C). Strikingly, while both groups showed gene upregulation compared to day 0, only the electrically stimulated myobundles exhibited a distinct set of 477 uniquely upregulated genes. Pathway analysis revealed significant enrichment in the Citric Acid (TCA) cycle, respiratory electron transport, and ATP synthesis (Fig. 5D) which are metabolic programs tightly associated with enhanced energy production and efficient muscle function. These findings suggest that, unlike unstimulated myobundles, electrically stimulated tissues undergo a metabolic switch consistent with endurance-like training, indirectly supporting a more advanced state of maturation. This aligns with previous reports showing that electrical stimulation fosters mitochondrial biogenesis, oxidative metabolism, and functional improvement in engineered muscle tissues [23,40].

### 3.6 Evaluating muscle contractility in fibrotic conditions: the role of fibroblasts and Nintedanib

Validated myobundles were used to simulate fibrotic conditions and analyze the impact of DMD fibroblasts on muscle contractility. First, human muscle fibroblasts were characterized through immunofluorescence, confirming that DMD fibroblasts exhibited increased expression of αSMA and Collagen I compared to healthy fibroblasts (Fig. S8, supplementary material). Morphologically, DMD fibroblasts were larger and exhibited a web-shaped appearance, in contrast to the spindle-like shape of healthy fibroblasts. Surface intensity maps also confirmed the increased expression of αSMA and Collagen I in DMD fibroblasts (Fig. S9, supplementary material). Co-culture models were created by integrating muscle fibroblasts into the myobundle in a single seeding process (Fig. 5E). First, two conditions were tested: muscle-only myobundles (HSMMs-only) and fibrotic muscle-fibro myobundles (F.DMD). To further demonstrate the potential of the model for drug discovery, we established a third condition (F.DMD + N) by treating the F.DMD model with 3 μM Nintedanib, in order to evaluate its anti-fibrotic effect, in line with previous in vitro dose–response studies on lung fibroblasts [41,42]. Nintedanib is an intracellular tyrosine kinase inhibitor that blocks pathways driving fibroblast proliferation and activation, thereby slowing fibrotic progression. It is approved for the treatment of idiopathic pulmonary fibrosis (IPF) [43], but has not yet been tested in the context of skeletal muscle fibrosis.

Immunofluorescence images illustrate differences in the expression of specific markers (SAA, F-actin, and FSP-1) across the three cultured 3D tissue conditions: HSMM, F.DMD, and F.DMD + N (Fig. 5F). To understand the intrinsic changes across the three different conditions, we performed a proteomic mass spectrometry analysis. Initially, we identified differences in the protein content among muscle-only myobundles (HSMM), fibrotic muscle-fibro myobundles (F.DMD), and fibrotic muscle-fibro myobundles treated with Nintedanib (F.DMD + N). From the results obtained, we generated a heatmap (Fig. 5G) revealing that key matrix proteins (e.g. LAMA4, LAMB4, ITGA2, ITGA3), which were higher in the F.DMD condition, were reduced by drug treatment in the F.DMD + N condition. Moreover, the matrix protein Tenascin-C (TNC), which is known to be significantly upregulated in fibrotic tissues and linked to organ fibrosis [44,45], exhibited the most markedly elevated level in F.DMD. Notably, Nintedanib treatment appeared to reduce TNC levels in the F.DMD + N condition compared to F.DMD, suggesting a potential antifibrotic effect of the drug on this glycoprotein. Furthermore, proteins associated with myofiber structure (e.g., MYL2 and MYOM1) and muscle contraction (e.g., TPM1, TPM2, and CASQ2) were found at lower levels in the F.DMD and F.DMD + N conditions compared to the HSMM condition (Fig. 5G). These findings suggest that dystrophic fibroblasts compromise the structural integrity of muscle tissue and potentially its functional contraction. However, the elevated protein levels associated with “abnormal skeletal muscle morphology” and “abnormal muscle physiology” pathways found in F.DMD vs. HSMM were reduced in the presence of Nintedanib (see Figs. S10 and S11, supplementary material). These results indicate that Nintedanib improves muscle bundle architecture compared to the untreated condition, though differences from the healthy control persist.

A second heatmap was generated from the proteomic analysis, specifically comparing the two fibrotic conditions (F.DMD and F.DMD + N) to assess the effects of Nintedanib treatment. This analysis examined protein expression differences between treated and untreated conditions, expressed as the ratio of F.DMD + N to F.DMD (F.DMD + N/F.DMD). The results showed decreased levels of matrix proteins (e.g., FN1, LAMB4) and increased levels of proteins associated with mitochondrial translation (e.g., MRPL30, MRPL43) and ATP generation (e.g., COX5B, NDUFS8) in the presence of Nintedanib. Furthermore, bar graphs depicting pathways associated with altered protein levels in F.DMD + N vs. F.DMD and F.DMD vs. HSMM illustrate that mitochondrial activity and ATP generation were reduced in the F.DMD condition compared to HSMM but appeared restored in the F.DMD + N condition relative to F.DMD (see Figs. S10–S11, supplementary material).

After a week of co-culture, myobundles were exposed to electrical stimulation and the force generated was optically quantified through the force-displacement relationship upon pillar bending (Fig. 5I). To measure the contraction force, both healthy control and fibrotic models were electrically stimulated for 24 h, and the resulting pillar bending was recorded. The presence of DMD fibroblasts reduced the force generated by the muscle constructs. Indeed, the force generated by the F. DMD model (6.6 ± 1.9 μN) was significantly lower than the HSMM-only model (20 ± 4.6 μN). Surprisingly, treating the fibrotic model with Nintedanib (F. DMD + N model) prevented the detrimental effect of DMD fibroblasts, resulting in a force generation (28.4 ± 9.4 μN) similar to the model without fibroblasts. These force measurements were coupled with quantifications of collagen content of the digested myobundles. Mirroring force measurements, we observed that DMD fibroblasts significantly increased the amount of collagen compared to any other condition. Remarkably, the treatment of Nintedanib reduced the collagen content, suggesting that collagen secretion might be a key factor in compromising the functionality of the myobundles and mimicking in vivo observations of pathological tissues [46] (Fig. 5J). Importantly, the proteomic signatures correlated with the functional outcomes of the myobundles. Increased ECM proteins (e.g., LAMA4, LAMB4, FN1, TNC) in F.DMD paralleled the higher collagen content and reduced force generation. Conversely, Nintedanib lowered ECM protein levels and partially restored mitochondrial and contraction-associated proteins (e.g., COX5B, MRPL30, MYL2, TPM1/2), consistent with the recovery of contractile force and reduced collagen deposition in F.DMD + N bundles.

To further support our findings, we performed independent co-culture experiments by incorporating healthy fibroblasts into our constructs, generating a F. healthy (HSMMs + healthy fibroblasts) model. Interestingly, the F. healthy model exhibited reduced force generation (9.97 ± 2.7 μN) compared to the HSMM-only model Notably, this reduction in force was not accompanied by an increase in collagen deposition (Fig. S12, supplementary material), suggesting that the impaired function cannot simply be attributed to fibrotic accumulation. Instead, these data indicate that the presence of fibroblasts, even in a non-pathological context, may modulate muscle contractility through mechanisms beyond extracellular matrix overproduction.

Overall, these experiments demonstrate that our model can be successfully used to perform functional screening of potential drugs (also repurposed) affecting the muscle microenvironment and modulating tissue contractility. It is important to highlight that the use of our novel setup based on horizontal pillars was critical to extract precise measurements of contraction force, which depends on the attachment point of the muscle bundles. This feature, neglected in current models, would be essential to generate reproducible quantifications and translate the obtained results into other laboratory settings.

### 3.7 Fibrotic and inflamed conditions promote EndoMT in the skeletal muscle model

EndoMT is a pathological mechanism involving EC trans-differentiation that compromises blood vessel structure and function. When associated with fibrosis, it explains how ECs lose their specific traits and acquire a fibrotic phenotype. This transition is characterized by the upregulation of αSMA, N-cadherin and other cytoskeletal or ECM-related proteins, along with the downregulation of EC identity markers like CD31 and VEGF receptors [47]. However, the relationship between EndoMT and other cellular populations within the muscle microenvironment remains poorly described. Additionally, the stimuli that drive EndoMT in skeletal muscle fibrosis and the timing of this transition, whether at the early stages of fibrosis or during a more established phase of the pathology, have yet to be identified. To evaluate the potential of the developed model for studying these cellular crosstalks, we recreated fibrotic and inflammatory conditions to investigate whether EndoMT can be induced by both microenvironments or if it is specifically dependent upon one of them.

**Fig. 6:**
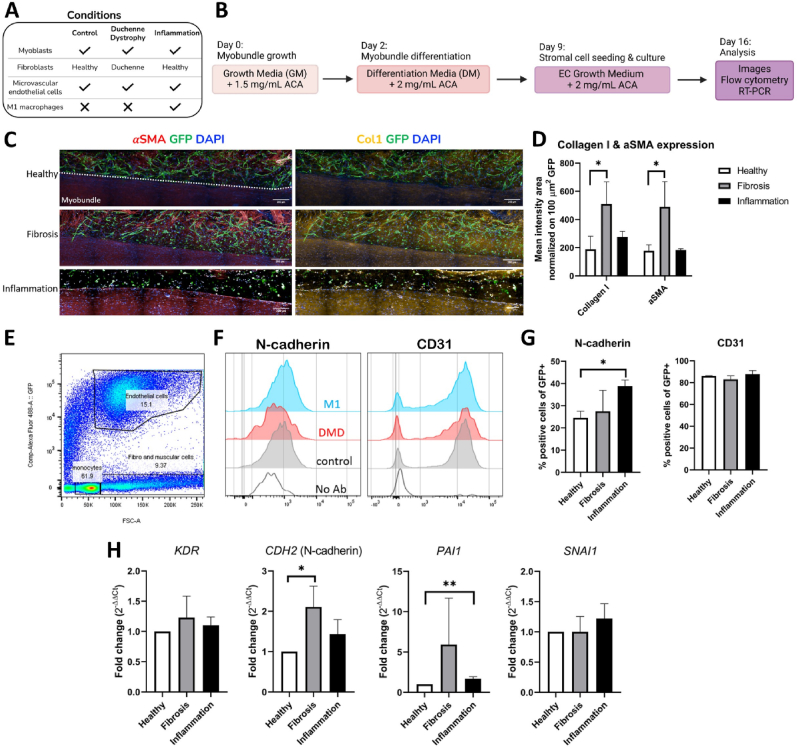
Analysis of the muscle microenvironment under fibrotic and inflamed conditions. A) Schematic of the tested conditions. B) Two-steps seeding procedure workflow. C) Representative images of co-cultured myobundles. Longitudinal sections, immunofluorescence image at day 14 of differentiation. Scalebar = 200 μm. D) Quantification of Col I and aSMA expression by ECs based on immunofluorescence images. Unpaired *t*-test performed to quantify statistical differences. ∗p < 0.05, N = 3. E) GFP + cells (endothelial cells) segregation by FACS. F-G) Flow cytometry quantification of N-cadherin and CD31 expression by GFP + cells (endothelial cells). ∗p < 0.05, N = 3. H) Gene expression analyses performed on sorted endothelial cells in Healthy, Fibrosis, and Inflammation models. Error bar is not present in Healthy models because their expression level was used to normalize the other conditions. ∗p < 0.05, ∗∗p < 0.01, N = 3.

Stromal cell populations were embedded in a fibrin gel and cast around differentiated myobundles to generate a 3D muscle microenvironment. Three different conditions were modeled: a fibrotic condition with DMD muscle fibroblasts; a control condition with healthy muscle fibroblasts; an inflamed condition with healthy muscle fibroblasts and M1-polarized macrophages (Fig. 6A). The culture period was divided into two phases (Fig. 6B). In the first step, HSMMs were seeded and differentiated to form the myobundle using differentiation medium for one week. In the second phase, stromal cells were seeded in a second hydrogel surrounding the myobundle, which was then cultured for an additional week with EGM-2MV medium. Immunofluorescence analyses were performed to quantify the expression level of αSMA and Collagen type I by ECs (Fig. 6C), being markers of fibrosis and EndoMT. The intensity of each marker was normalized to the area of cytoplasmic GFP expressed by ECs to accurately quantify the protein signal produced by ECs.

Both αSMA and Collagen type I significantly increased in the Duchenne fibrosis model compared to the healthy control group (490 ± 146.05 vs. 178 ± 34.29 for αSMA and 509.67 ± 129.16 vs. 189 ± 75.51 for Collagen I, normalized mean intensity area) (Fig. 6D). In contrast, the inflamed microenvironment did not exhibit any significant difference in the expression of these markers (183 ± 8.16 (αSMA) and 273.33 ± 33.41 (Collagen I)) when compared to the same control (Fig. 6D). Significantly higher was also αSMA and Collagen I expression by the rest of the tissue in the fibrosis condition markers (9933.13 ± 5859.80 (αSMA) and 9574.78 ± 5498.30 (Collagen I)) with respect to the healthy one markers (5731.05 ± 1739.31 (αSMA) and 4732.3 ± 1079.07 (Collagen I)) (Fig. S13, Supplementary Material). Moreover, the inflamed condition prevented the formation of vascular networks (Fig. 6C). Conversely, a clear formation of branched vascular network was observed in the control condition and surprisingly also in the fibrotic one (Fig. 6C). Network analysis performed on the different 3D models showed higher network density and total branch length in healthy condition with respect to fibrotic and inflamed samples. Regarding network area, no difference was observed comparing heathy and fibrotic conditions (Fig. S14, Supplementary Material).

To further evaluate potential phenotypical changes in ECs due to activation of EndoMT, we first performed enzymatic digestion of the hydrogel to retrieve the cells, followed by fluorescence-based cell sorting to isolate GFP-positive ECs (Fig. 6E). The mean expression of the EC identity marker CD31 was 86.1 %, 83 %, and 87.8 % in the healthy, fibrosis, and inflammation conditions, respectively, confirming the successful retrieval of a purified EC population for subsequent experiments [48]. A preliminary analysis was then performed through flow cytometry to quantify the expression level of N-cadherin, being an established marker of EndoMT [47] (Fig. 6F–G and Fig. S15, supplementary material). In the inflammation model, 39 ± 2.7 % of GFP + cells resulted positive for N-cadherin, compared to 27.5 ± 9.3 % and 24.5 ± 3 % within the fibrosis and healthy models, respectively (Fig. 6G).

The viability of M1 macrophages was assessed after one week of co-culture, showing a viability of 70 ± 7 %, which was comparable to the average viability of GFP + cells (70 ± 10 %). Notably, the presence of M1 macrophages did not impact GFP + cell viability, as 77.8 ± 9 % of GFP + cells remained viable in the co-culture, closely matching the 77.93 ± 7 % viability observed in the healthy control without M1 macrophages.

Moreover, sorted ECs were analyzed using RT-qPCR to assess the expression of a panel of genes strongly associated with EndoMT. The selected markers included *CDH2* (N-cadherin), *KDR*, *PAI1*, and *SNAI1* (Fig. 6H). Interestingly, these molecular analyses revealed a shift in gene expression related to EndoMT. Specifically, we observed an upregulation of *CDH2* (N-cadherin) in the fibrosis model and a positive trend in the inflammation model. Moreover, an increase in *PAI1* expression was also observed in both the inflammation and fibrosis models. At the same time, no significant change was detected in the expression of the mesenchymal gene *SNAI1* and of the EC identity gene *KDR*. Overall, these results highlight that our model was able to capture an early signature of EndoMT in the skeletal muscle microenvironment and to extract peculiar structural and molecular features associated with both fibrotic and inflamed conditions.

## 4 Discussion

Skeletal muscle exhibits a unique 3D architecture with aligned, multinucleated myofibers capable of force generation, surrounded by a fibroblast-rich endomysium and intertwined with microvascular networks forming the muscle microenvironmen [49]. This spatial organization is crucial for muscle function. However, existing 3D in vitro muscle models predominantly focused on developing muscle fibers or bundles, often neglecting the integration of a comprehensive stromal compartment [[50], [51], [52]]. Indeed, previous studies either lacked specific cell types typically found in physiological muscle tissue, such as fibroblasts [53], or did not develop complex vascular trees allowing to characterize the interactions between microvascular networks and the muscle microenvironment [54]. A previous work provided a more realistic model of the complex muscle microenvironment, involving differentiated human muscle fibers surrounded by a fibroblasts-rich endomysium and a microvascular network integrated with mural-like cells [32]. However, no demonstration of muscle contractility was provided and potential fibrosis-induced molecular changes in the stromal compartment were not evaluated.

The limited availability of muscle models that recapitulate the heterotypic interactions of the muscle microenvironment has hindered progress in identifying agents that neutralize or reverse fibrosis in muscular dystrophies and chronic myopathies. To address this gap, we leveraged 3D biofabrication techniques to recreate a mesoscale human skeletal muscle microenvironment incorporating both connective and vascular compartments, thereby closely mimicking native tissue. Building on previous models also developed by our lab [32,35,55], we designed a novel mesoscale system in which 3D muscle constructs are anchored between two cylindrical PDMS pillars, providing structural support and ensuring proper myobundle stretching. While pillar-based setups have been previously used [19,22,36], our model is among the largest of its kind. Indeed, the engineered construct measures 6 mm in length and 1 mm in diameter, comparable to models by Vann et al. (7 mm × 2 mm) [56] and Maffioletti et al. (8 mm × 1 mm) [28], and significantly larger than those by Shimizu et al. (3 mm × 0.5 mm) [50] and Rose et al. (150 μm × 50 μm) [52]. Moreover, functional contractile properties have been investigated with a post-deflection approach [57,58], which was applied to a novel horizontal pillar arrangement [22,37,55,59].

Most studies use a force calculation method that considers pillar bending as a fixed linear elastic cantilever beam with a concentrated load applied at the free end [21]. To better reflect the practical bending behavior of the pillars, it is more accurate to account for a uniformly distributed load across the myobundle thickness attached to the pillar, rather than assuming a concentrated load at the free end. In this study, our innovative setup improved the accuracy of force estimation by precisely quantifying the distance between the fixed base and the myobundle attachment point.

We performed analyses to confirm that the muscle constructs exhibited key structural and functional characteristics by comparison with previously published works [58]. In this regard, we showed that the model significantly promoted the formation of highly aligned multinucleated myofibers resulting in pre-tensioned myobundles. The expression of SAA and its typical banding further confirmed the high level of maturation achieved by the myobundles. Obtaining this uniform alignment is important for the final architecture of the biofabricated muscle, as it supports maximal force generation during contraction upon electrical stimulation [60].

Gene expression analysis of the non-stimulated samples at day 14 compared to day 0 showed significant upregulation of the TPM1 sarcomeric marker gene, which is specific to Type-IIa muscle fibers. However, there was a much greater presence of Type-I specific muscle fibers, such as TNNC1, TNNI1, TNNT1, MYH7, MYL2, MYL3, and calcium transport marker genes like CASQ2. Overall, this suggests that the myobundles in our muscle model are predominantly composed of Type-I muscle fibers [61]. Also known as Slow Oxidative (SO) fibers, these fibers are characterized by their relatively slow contractions and reliance on aerobic respiration to generate ATP. They produce low-power contractions over extended periods and are resistant to fatigue.

A key feature of our model is the presence of a customized electrical stimulation setup, capable of delivering pulses to up to 36 independent myobundles. The stimulation platform was microfabricated with standard multiwell dimensions, ensuring compatibility with closed microscope systems. This represents a fundamental step toward scaling out the model and enabling high-throughput drug screening. For electrical stimulation, we adopted a long-term intermittent regime as in previous studies [23,24,62]. By testing multiple stimulation patterns, we identified a regime which, unlike earlier applications [24], enabled simultaneous contraction of multiple fibers across the entire 3D tissue, producing active contractions synchronous with the stimulation frequency. This stimulation generated pillar bending and force peaks of over 20 μN at 1 Hz. These forces were comparable to values reported in other studies, with expected variation due to differences in force measurement methods, construct dimensions, or cell sources [22,23,37,39,50,62]. It is important to emphasize that the presence of our novel pillar setup allowed precise quantifications that take into account the exact attachment point of each myobundle. Although the attachment position on the pillar varied slightly between myobundles, direct imaging (Fig. 4C) enabled us to determine the mean attachment height and thereby quantify with high accuracy the force exerted at that specific location (Fig. 2F).

We took advantage of the custom stimulation setup to analyze the impact of electrical stimulation on the molecular profile of the myobundles. We identified a list of genes uniquely upregulated in presence of electrical stimulation compared to samples at day 0, observing an enrichment for biological processes that include respiratory electron transport and ATP synthesis. This result suggests a higher energy request from stimulated myobundles, which is essential to promote tissue maturation during training. Furthermore, from the same enrichment analysis we found that “inflammatory response” and “myc targets” were negatively enriched in electrically stimulated samples. This result prompts us to argue that there is less inflammation in presence of stimulation and less activation of the Myc transcription factor, which inhibits myoblast differentiation [63].

Overall, these results highlight the critical role of biophysical stimulation in muscle maturation and systemic contraction, which is essential for linking dysregulated molecular pathways to functional outcomes.

Beyond generating functional muscle bundles, our study incorporated a stromal compartment to directly assess the effects of fibrotic ECM and stromal cells on muscle tissue, an aspect overlooked in previous models. We simulated fibrotic conditions by co-embedding muscle myoblasts and fibroblasts within the same hydrogel, allowing us to assess the impact of fibroblasts on muscle contractility and the response of our fibrotic model to the anti-fibrotic drug Nintedanib [41].

Proteomic analysis revealed that Nintedanib treatment moderately reduced levels of matrix proteins such as laminin and fibronectin, as well as the fibrosis-associated protein Tenascin-C (Fig. 5G and H).

Notably, limiting the production of these proteins has been shown to help mitigate and attenuate organ-specific fibrosis [64,65].

Additionally, results indicated that dystrophic fibroblasts disrupt muscle tissue integrity and impair functional contraction (Fig. S11). However, Nintedanib treatment partially reversed these effects, improving skeletal muscle morphology compared to the untreated condition (Figs. S10–S11). Furthermore, contraction force quantification confirmed that DMD fibroblasts impair force generation, whereas Nintedanib treatment restored this function in the F.DMD model, leading to increased force generation (Fig. 5I).

Multiple factors are known to influence muscle contraction outcomes, including pillar stiffness, myobundle size, cell source, electrode material, stimulation pattern and force measuring system. These parameters present a high variability throughout the different studies and make it difficult to compare our contractile performance across the different experimental platforms, which differ in more than one experimental set-up. To investigate how our models respond to variations in electrical stimulation, we conducted a proof-of-concept study using 99.5 % pure platinum electrodes in a newly designed set-up aligned with myobundles. Peaks of absolute forces were recorded as 44.73 μN, 12.61 μN, and 21.91 μN for the HSMM, F.DMD, and F.DMD + N conditions, respectively (see Fig. S16, supplementary material) with values of the healthy control comparable with similar studies [39].

Using the cross-sectional area of the myobundles, the specific contraction force was also determined with peaks of 341.13 μN, 71.58 μN, and 150.39 μN for the HSMM, F.DMD, and F.DMD + N conditions, respectively (see Fig. S17–S18, supplementary material). Overall, the quantified contraction forces exhibited a response trend consistent with our previous findings (see Fig. 5H), but with much higher force intensities.

The deleterious effect of DMD fibroblasts may be attributed to the overproduction of collagen, while Nintedanib could interfere with collagen synthesis, as demonstrated in lung fibrosis [66]. Thus, we hypothesized that varying collagen levels in the biofabricated myobundles could explain these results. To test this hypothesis, we quantified the secreted collagen content using a colorimetric assay. The F. DMD model exhibited a significantly higher collagen content, particularly differing from both the HSMM only and the fibroblast-DMD + Nintedanib (F. DMD + N) models. This result suggests that the Nintedanib treatment on the F. DMD model could limit collagen accumulation, aligning with previous studies [41,43].

Overall, protein mass spectrometry, collagen quantification, and force measurements demonstrated that DMD fibroblasts increase collagen deposition in the myobundle, disrupting muscle fiber structure and limiting force transmission to the anchoring system. More importantly, we assessed how Nintedanib treatment mitigates these deleterious effects, leading to improved muscle structure, reduced collagen deposition, and enhanced force exertion.

These findings confirmed that the presence of Duchenne-derived fibroblasts increased collagen content in the tissue, thereby impairing the force capacity of fibrotic muscles. This relationship is supported by other studies suggesting that muscle stiffness is primarily related to the presence of ECM, and an excessive ECM amount can lead to pathological stiffness [67]. The skeletal muscle ECM is predominantly composed of elongated collagen fibrils, which can be oriented differently relative to the direction of contraction. ECM stiffness in the muscle-tendon junction is positively correlated with muscle force because it plays a fundamental role in force transmission. However, when ECM accumulates in the main body of the muscle, it acts as a passive element which reduces the displacement induced by contraction force [68]. Indeed, in our study DMD fibroblasts caused an increased amount of collagen dispersed in the myobundle, limiting force transmission to the anchoring system.

Finally, we employed our model to analyze EndoMT-related molecular changes occurring within the vasculature in the fibrotic and inflammatory microenvironment characteristic of muscular dystrophies. In the last few years several studies have focused on proving the prominent participation of EndoMT in the generation of activated myofibroblasts during the development of tissue fibrosis occurring in kidney [69,70], liver [71,72] and cardiac tissues [[73], [74], [75]]. However, the biological mechanisms that generate EndoMT are yet to be fully elucidated. Moreover, no studies attempted to translate what has been observed in other tissue-specific fibrosis to the context of skeletal muscle and no in vitro 3D models were developed to analyze this process.

Previous co-culture approaches combining 3D muscle tissues with endothelial cells mainly aimed to study microvascular growth and its effects on muscle structure and function [30,31,76], leaving EndoMT largely unexplored. Here, we used our skeletal muscle model to expose ECs to fibrotic and inflamed microenvironments, aiming to better understand the conditions that promote EndoMT.

We recreated three different conditions: a pathological model incorporating DMD-derived muscle fibroblasts, a control condition with healthy muscle fibroblasts, and an inflammatory-like condition combining healthy muscle fibroblasts and M1-polarized macrophages. In all cases, these cells were embedded in the fibrin gel surrounding the myobundles together with ECs. This setup enabled the formation of microvascular networks around the muscle bundle.

We combined confocal imaging, flow cytometry, and RT-qPCR to identify signatures of EndoMT. Our results showed an increase in αSMA and Collagen I in ECs co-cultured with DMD fibroblasts. Moreover, gene expression analyses on retrieved ECs showed an upregulation of *CDH2* and *PAI1* (associated with EndoMT) in the fibrosis model and in the inflammation model, respectively. Additionally, retrieved ECs analyzed through flow cytometry were positive for the N-cadherin marker in the inflamed condition compared to the control. Interestingly, the expression of the EC identity marker CD31 remained above the 80 % threshold for all conditions. Similarly, no significant difference was quantified in the gene expression analysis of the *KDR* gene (encoding VEGFR-2) across the three conditions.

Overall, since the expression of CD31 and VEGFR-2 in ECs is known to decrease during EndoMT [8], these results suggest that EC identity was maintained in all conditions and that we were observing the early phases of trans-differentiation to a mesenchymal phenotype that can potentially affect the whole muscle microenvironment. Finally, it is important to highlight that both DMD fibroblasts and M1 macrophages can influence ECs to adopt a pro-fibrotic phenotype.

Although no previous in vitro 3D models analyzed the role of EndoMT on skeletal muscle, our findings align with studies on EndoMT occurring in other tissues. For example, Alonso-Herranz and colleagues cultured mouse cardiac ECs and fibroblasts with bone marrow-derived macrophages or supplemented with TGFβ, caused the downregulation of EC-related genes (Pecam, Kdr) and the upregulation of EndoMT markers like Serpine1 (Pai1) in both conditions [77]. Yi et al. used a co-culture system allowing direct contact between human fetal lung fibroblasts and irradiated ECs. Interestingly, radiation-induced EndoMT in ECs, significantly increasing Snail expression and reducing CD31 expression [78].

Future work may address the current limitations of the model. For instance, it would be valuable to confirm the stiffness increase of the DMD-fibrotic model by quantifying the load stiffness and assessing its relationship to the reduced contraction force and increased matrix stiffness. The potential influence of mural cell types such as pericytes, known to have a role in fibroblast activation and fibrogenesis [79,80], should also be investigated. Building on this, more complex co-culture systems could be established, similar to our previously published work [32] and assess whether the presence of pericytes could generate myofibroblasts or enhance EndoMT and stimulate muscle fibrosis in our 3D muscle tissue. Furthermore, the phenotypical changes observed in ECs were only partial, likely due to the short timeline of the experiments. Hence, a prolonged co-culture could lead to more pronounced pathological changes in the endothelial phenotype. Finally, starting from the results here presented, it would be relevant to combine different omic datasets and perform mechanistic studies to identify potential therapeutic solutions for EndoMT. This would require testing various concentrations and combinations of drugs, thus needing a more high-throughput platform. Nintedanib has demonstrated to suppress EndoMT in vivo using models of Bleomycin-induced pulmonary fibrosis [81]. Hence, testing the effects of Nintedanib at multiple dosage and exposure times could help to understand if it shows anti-EndoMT effects also in the context of muscle fibrosis. To conclude, evaluating the efficacy of additional anti-fibrotic agents would be essential to fully validate the fibrotic model. Given the mechanistic diversity of anti-fibrotic compounds (e.g., TGF-β inhibitors, Platelet-Derived Growth Factor (PDGF) inhibitors, Pirfenidone) it is crucial to assess therapeutics targeting distinct molecular pathways to capture a broader spectrum of potential therapeutic effects.

In conclusion, this novel in vitro model holds promise for advancing our understanding of the biological and pathological mechanisms involved in muscle fibrosis, highlighting the potential role of endothelial dysfunction in disease progression. The model combines key elements, such as contractile myobundles and stromal cells, to create a complex 3D microenvironment of human skeletal muscle that has not been previously documented. As a result, these bioengineered myobundles offer a novel platform for investigating skeletal muscle function across numerous physiological and pathological scenarios. Importantly, recapitulating EndoMT in a human skeletal muscle context opens translational avenues for identifying early fibrotic signatures, discovering potential biomarkers, and screening antifibrotic therapies [82]. Our findings with Nintedanib illustrate how this platform can support preclinical drug evaluation, and future incorporation of patient-derived cells may extend its use toward personalized therapy in muscular dystrophies and chronic myopathies.

## CRediT authorship contribution statement

**R. Francescato:** Writing – review & editing, Visualization, Methodology, Formal analysis, Conceptualization. **M. Ishmaku:** Writing – review & editing, Writing – original draft, Visualization, Methodology, Formal analysis. **G. Talò:** Supervision, Methodology. **M. Francese:** Methodology. **L. Cascione:** Formal analysis. **V. Martini:** Methodology, Formal analysis. **M. Uguccioni:** Supervision, Conceptualization. **M. Moretti:** Writing – review & editing, Supervision, Funding acquisition, Conceptualization. **S. Bersini:** Writing – review & editing, Supervision, Funding acquisition, Conceptualization.

## Declaration of competing interest

The authors declare that they have no known competing financial interests or personal relationships that could have appeared to influence the work reported in this paper.

## Data Availability

Data will be made available on request.
