# Supplementary material for: Biofabrication of a 3D human skeletal muscle microenvironment to study the early steps of fibrosis
Source: Mater Today Bio. 2025 Oct 15;35:102386. doi: 10.1016/j.mtbio.2025.102386 (PMC12554888; doi:10.1016/j.mtbio.2025.102386)
Supplement: Multimedia component 2 [file mmc2.docx]

**SUPPLEMENTARY MATERIAL**


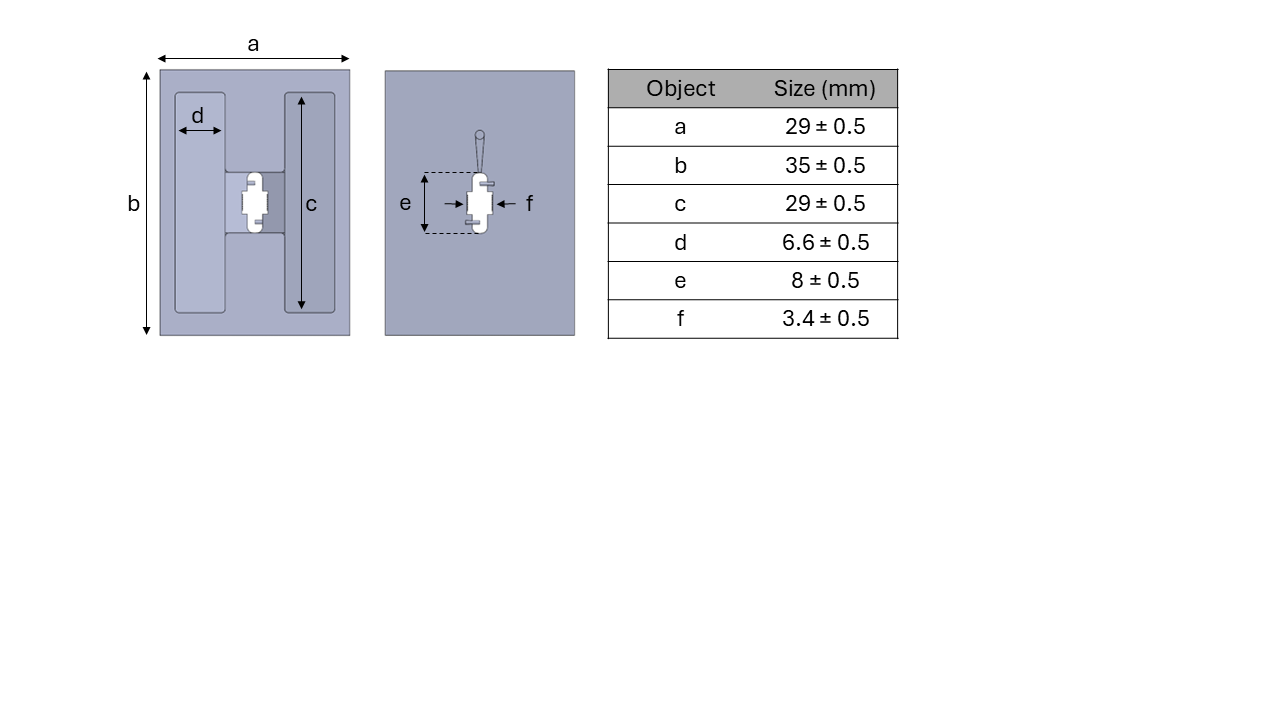


*Figure S1. Dimensional annotations of the PDMS structure.*


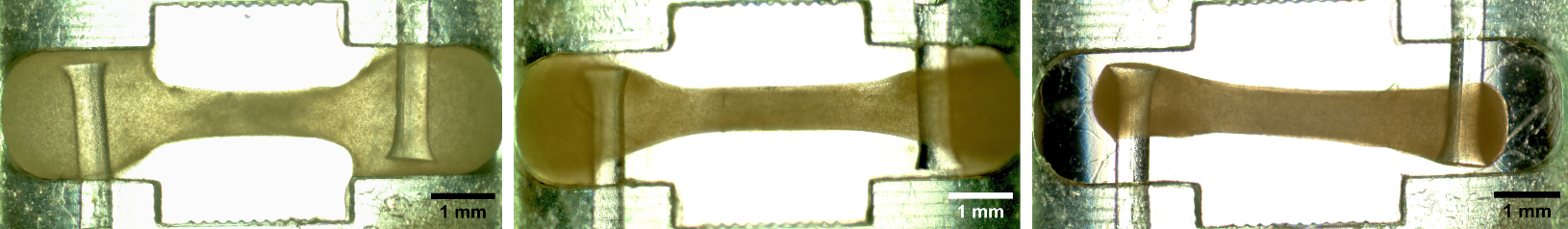


Figure S2. (from left) Brightfield images of the myobundle at day 1, 3 and 7 of maturation. Scalebar: 1 mm.


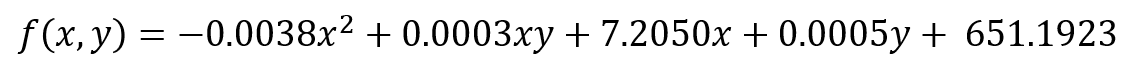


Figure S3. Second-order polynomial function from mechanical characterization and computational simulation.


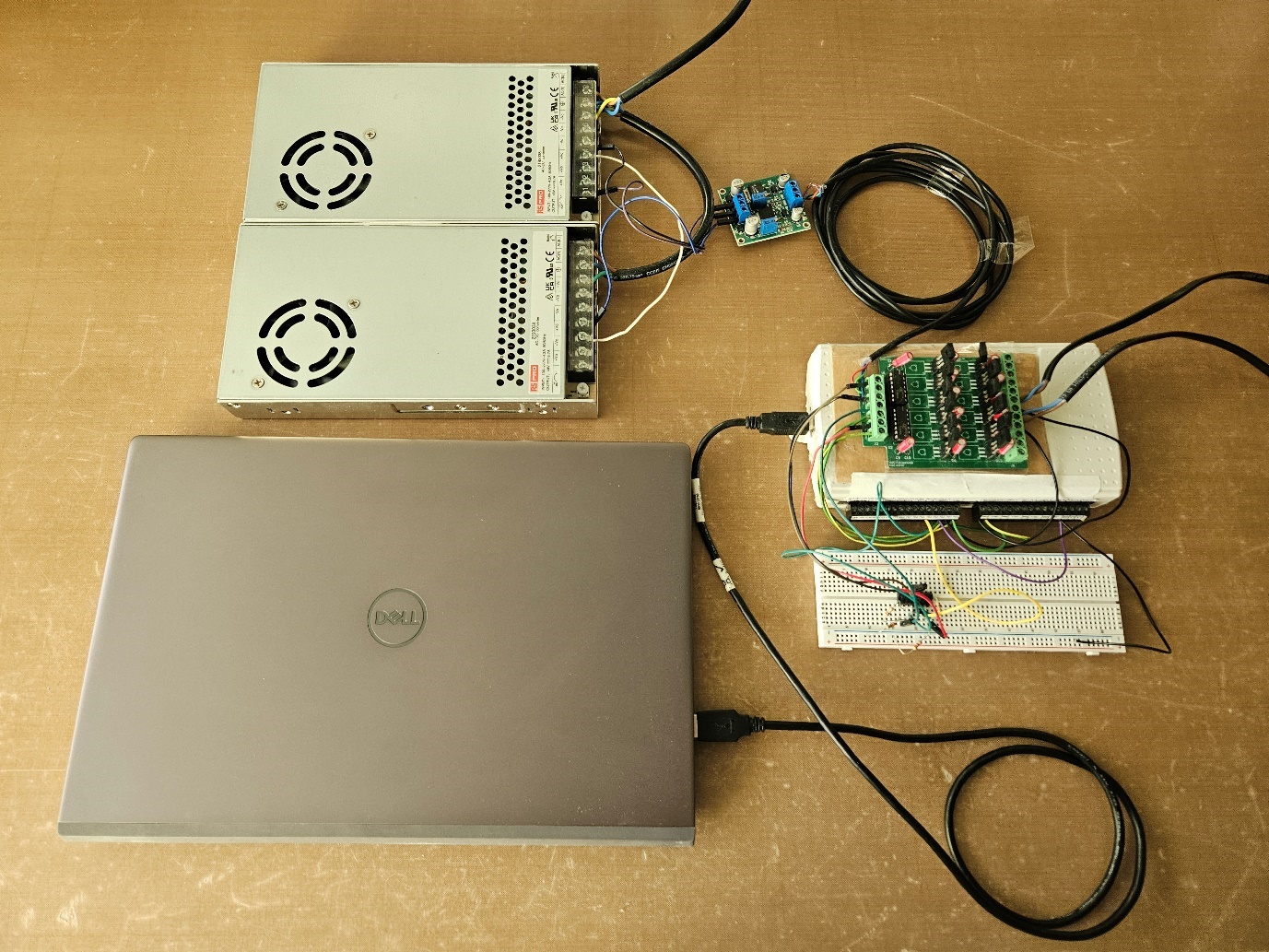


**CONTROL BOARD**

**DUAL POWER SUPPLY**

**PC**

**AMPLIFICATION UNIT**

Figure S4. Picture of the electrical stimulation system composed of a PC, dual voltage power supply, control board and amplification unit.


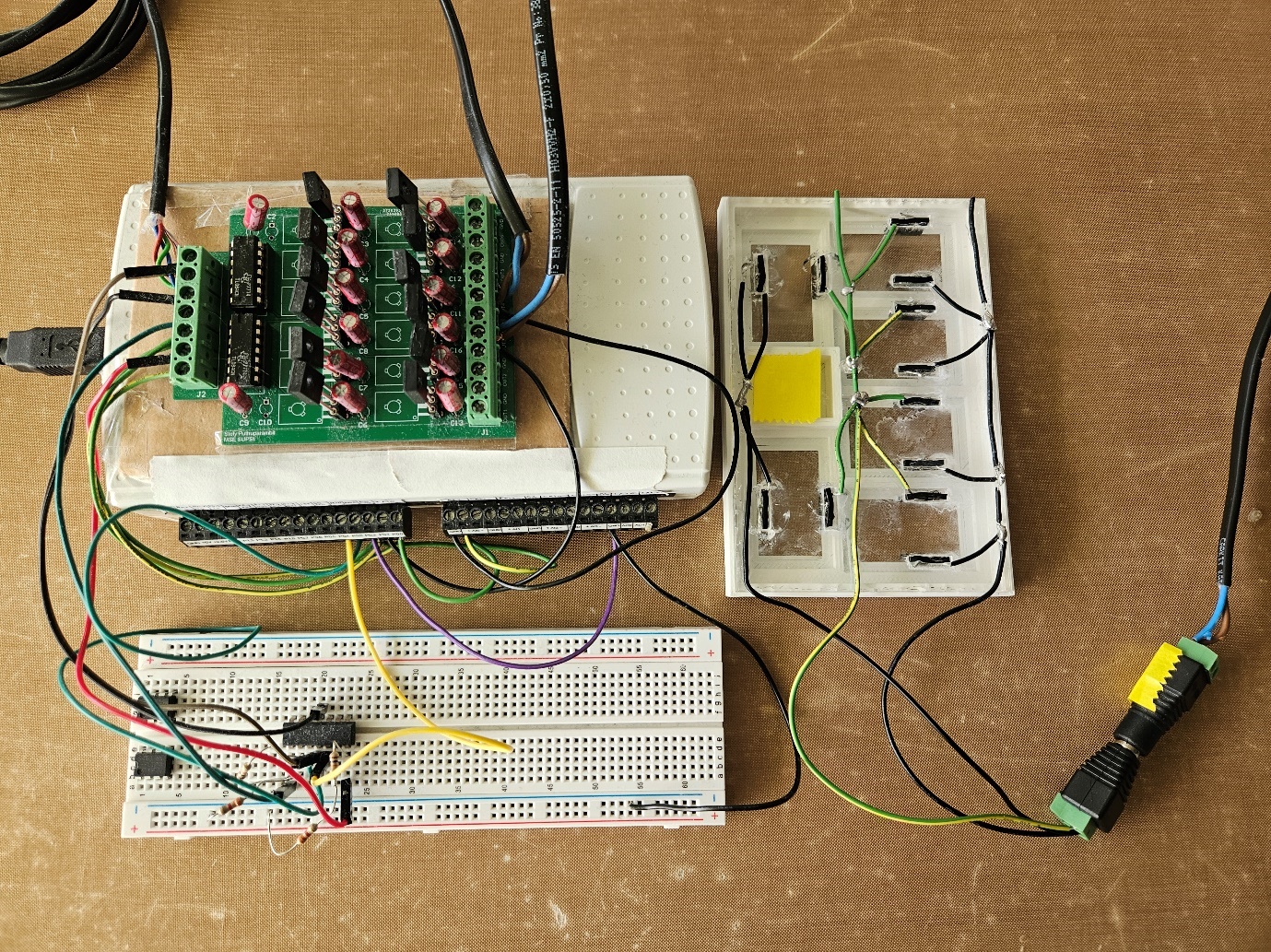


**STIMULATION PLATFORM**

Figure S5. Picture of the control board, amplification unit and the attached platform.


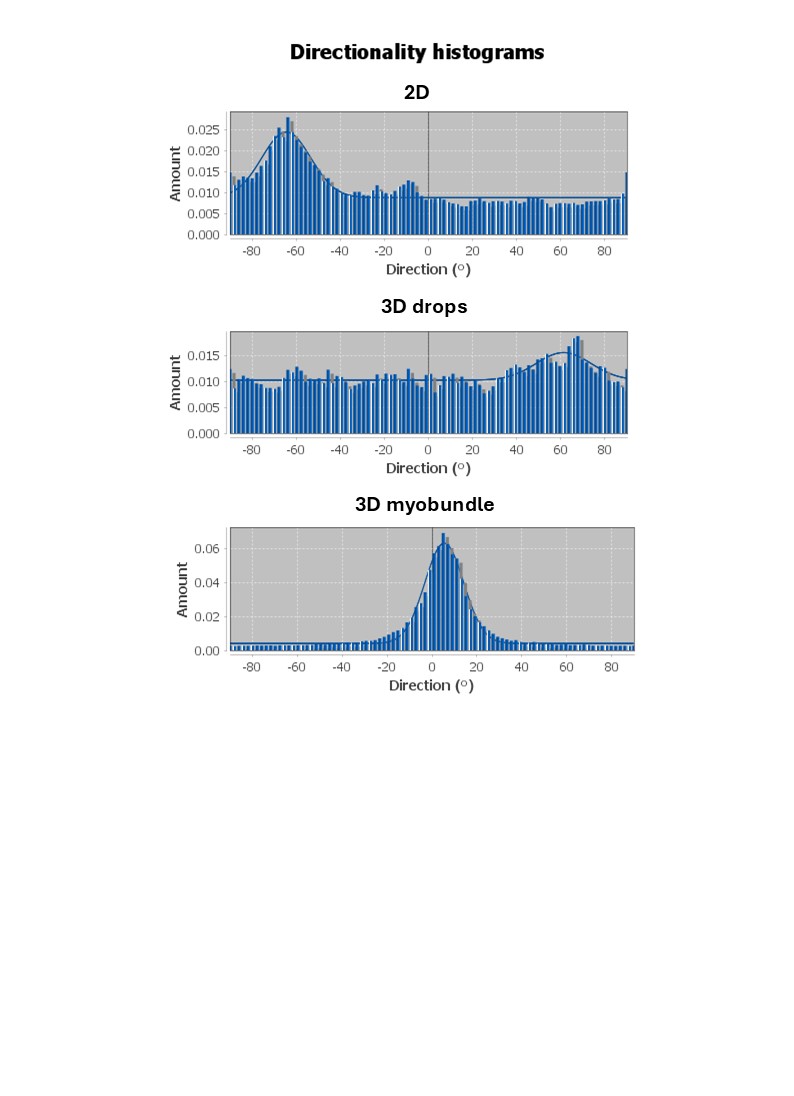


Figure S6. Myotube alignment assessment: directionality histograms and Gaussian fit in the 2D, 3D drops and 3D myobundle culture condition. Histograms illustrate the distribution of orientation angles (degrees from the horizontal axis) for each condition.


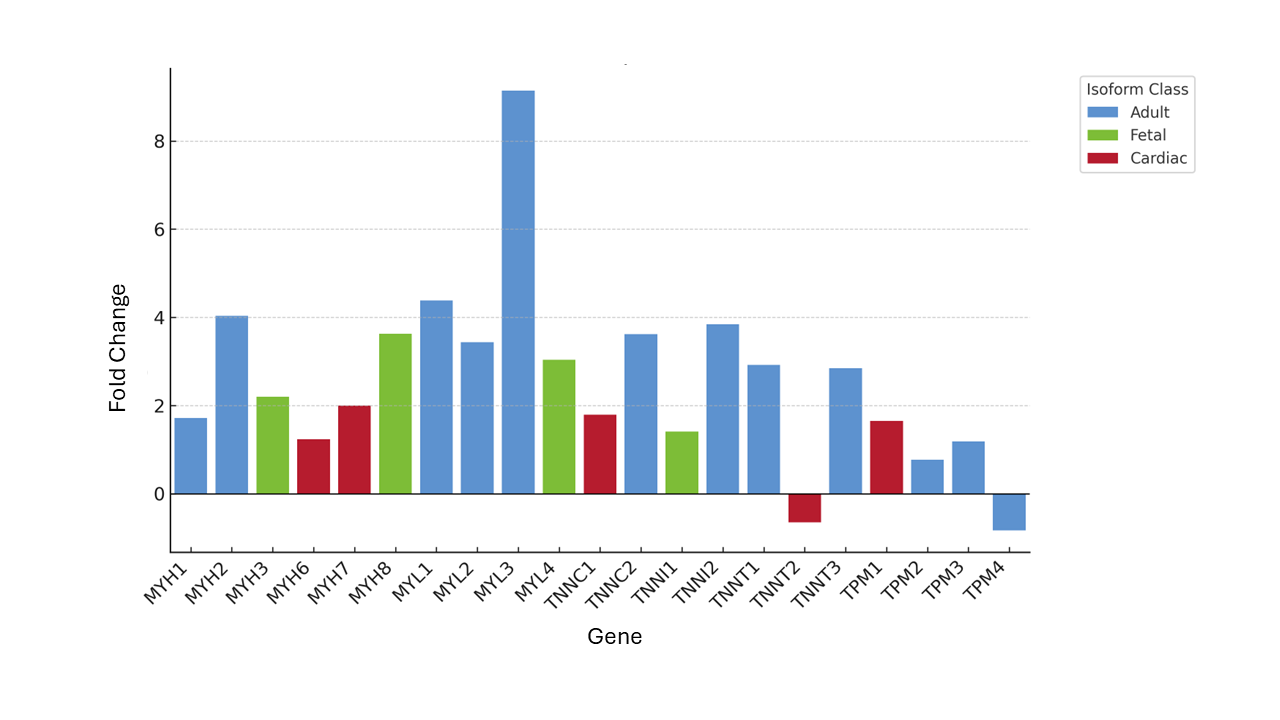


Figure S7. Expression of contractile protein isoforms in biofabricated myobundles (D14 vs D0, non-stimulated). Bar plot shows the Fold Change (logFC) of selected contractile isoforms, calculated as Day 14 relative to Day 0. Positive values indicate upregulation at Day 14. Isoforms are color-coded by class: Adult (blue), Fetal (green), and Cardiac (red).


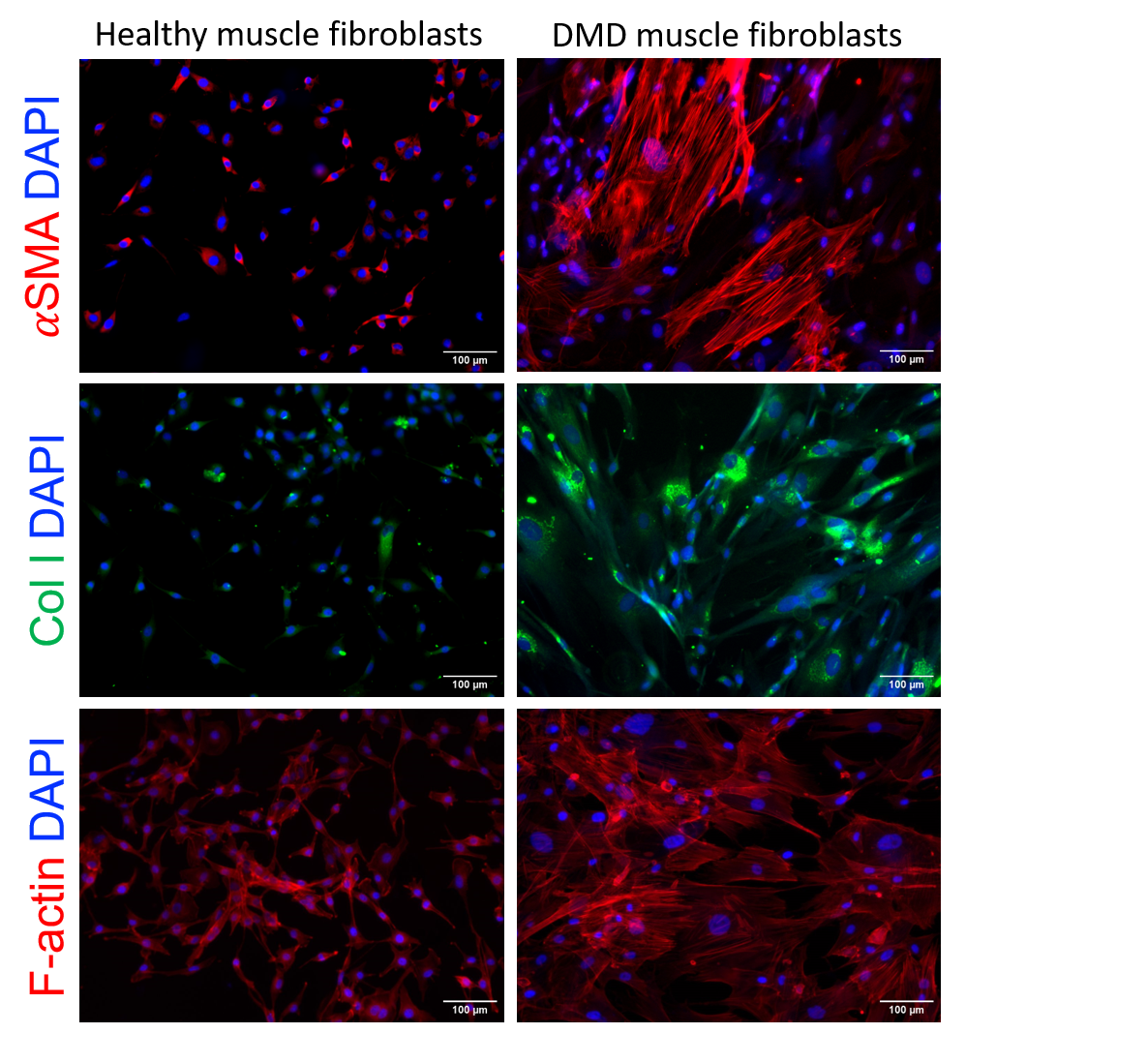


Figure S8. Immunofluorescence characterization of healthy and DMD fibroblasts showing an increased expression of Collagen I and αSMA in DMD fibroblasts. (Scalebar: 100 μm)


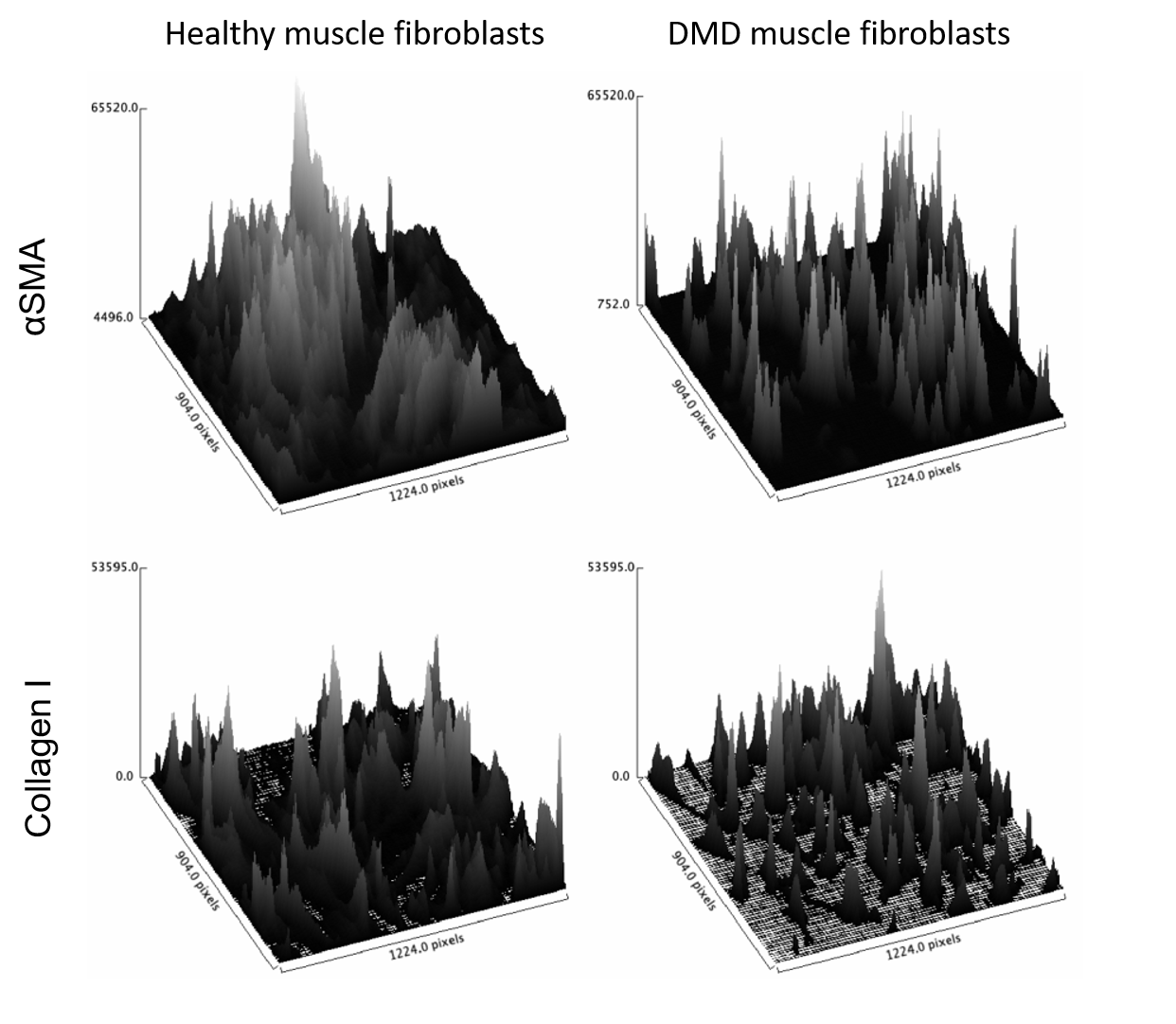


Figure S9. Surface plots of the immunofluorescence characterization of healthy and DMD fibroblasts. Intensity maps of expression ofαSMA (top) and Collagen-1 (bottom) are shown.


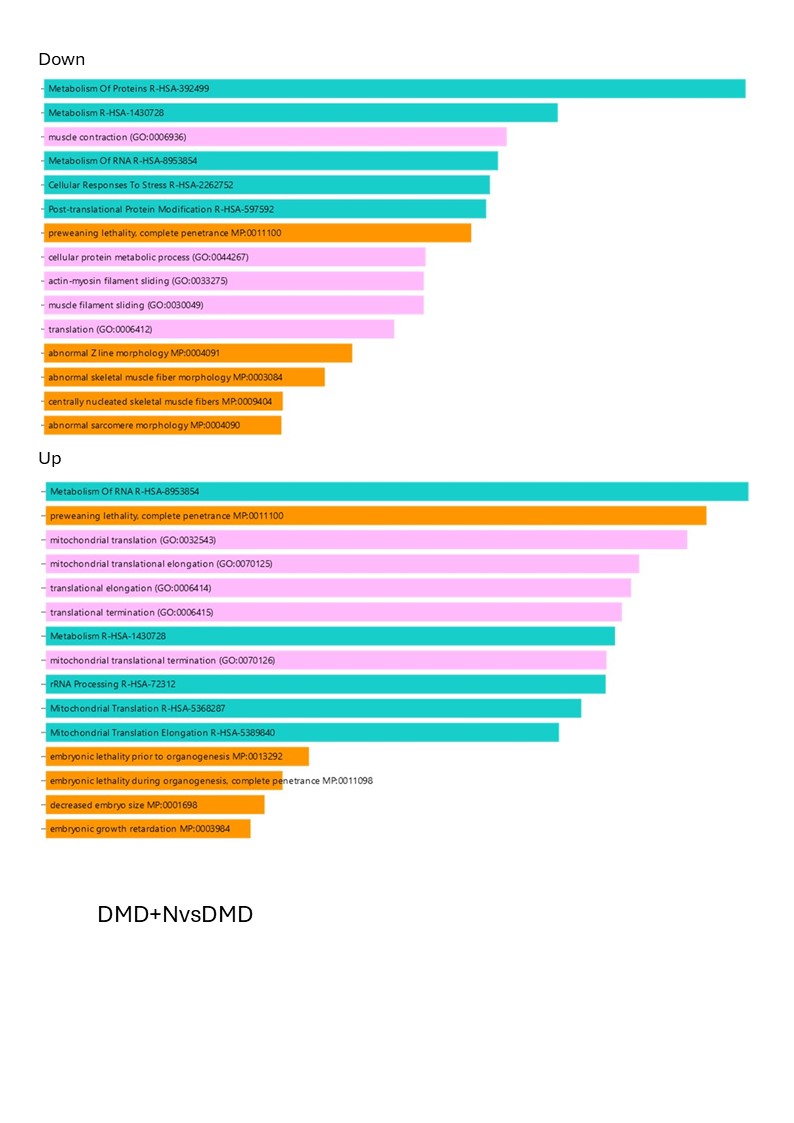


Figure S10. Bar-graph illustrating pathways and processes associated with decreased and increased protein levels in the F.DMD+N condition relative to F.DMD.


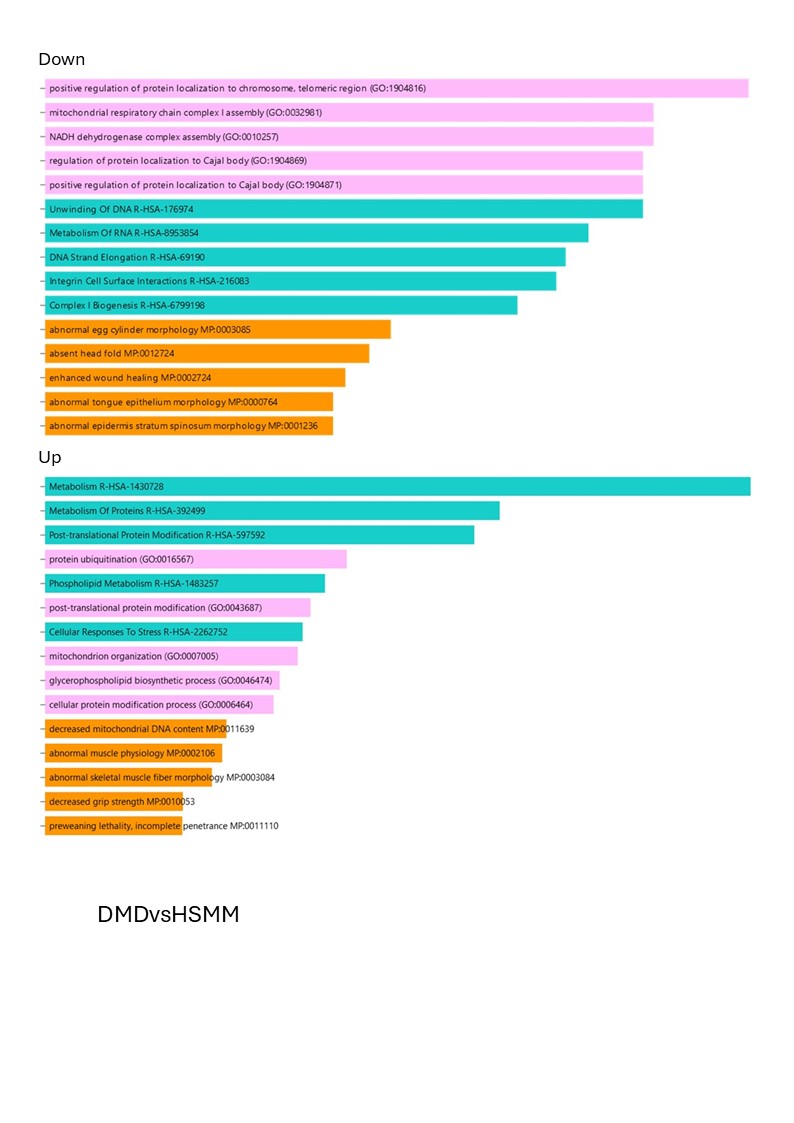


Figure S11. Bar-graph illustrating pathways and processes associated with decreased and increased protein levels in the F.DMD condition relative to HSMM.


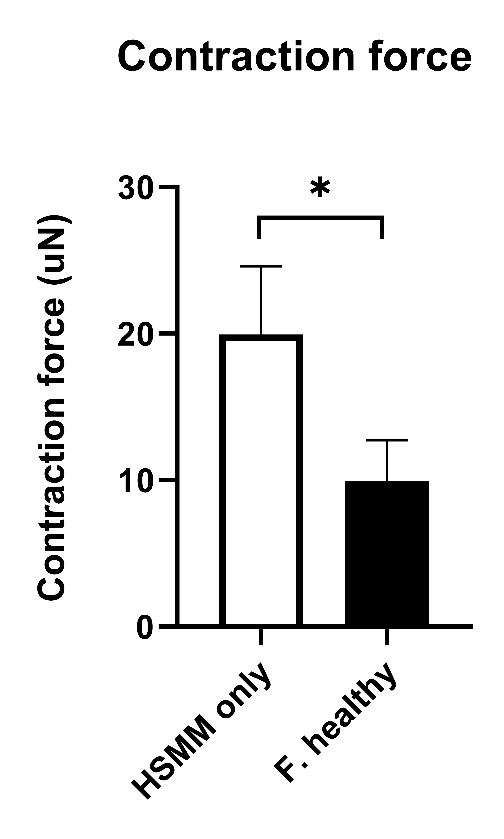

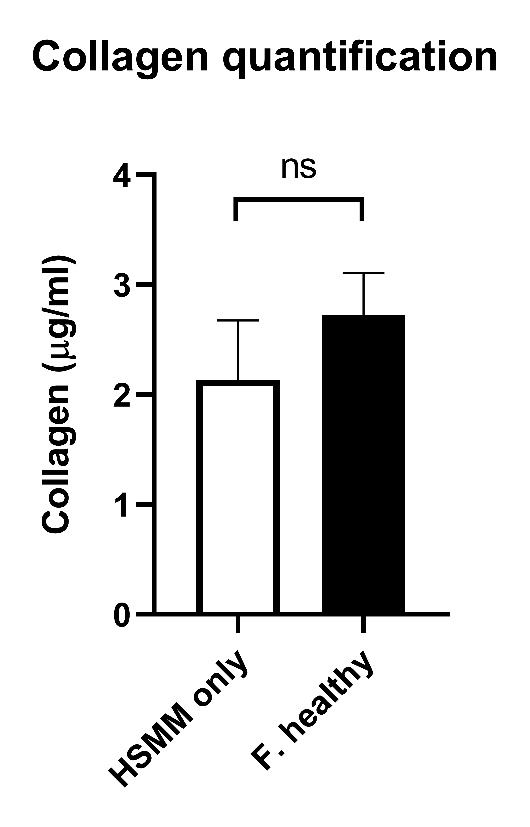


Figure S12. Contraction force quantification of only muscle and healthy muscle-fibro myobundles and collagen quantification in only muscle and healthy muscle-fibro myobundles culture conditions.


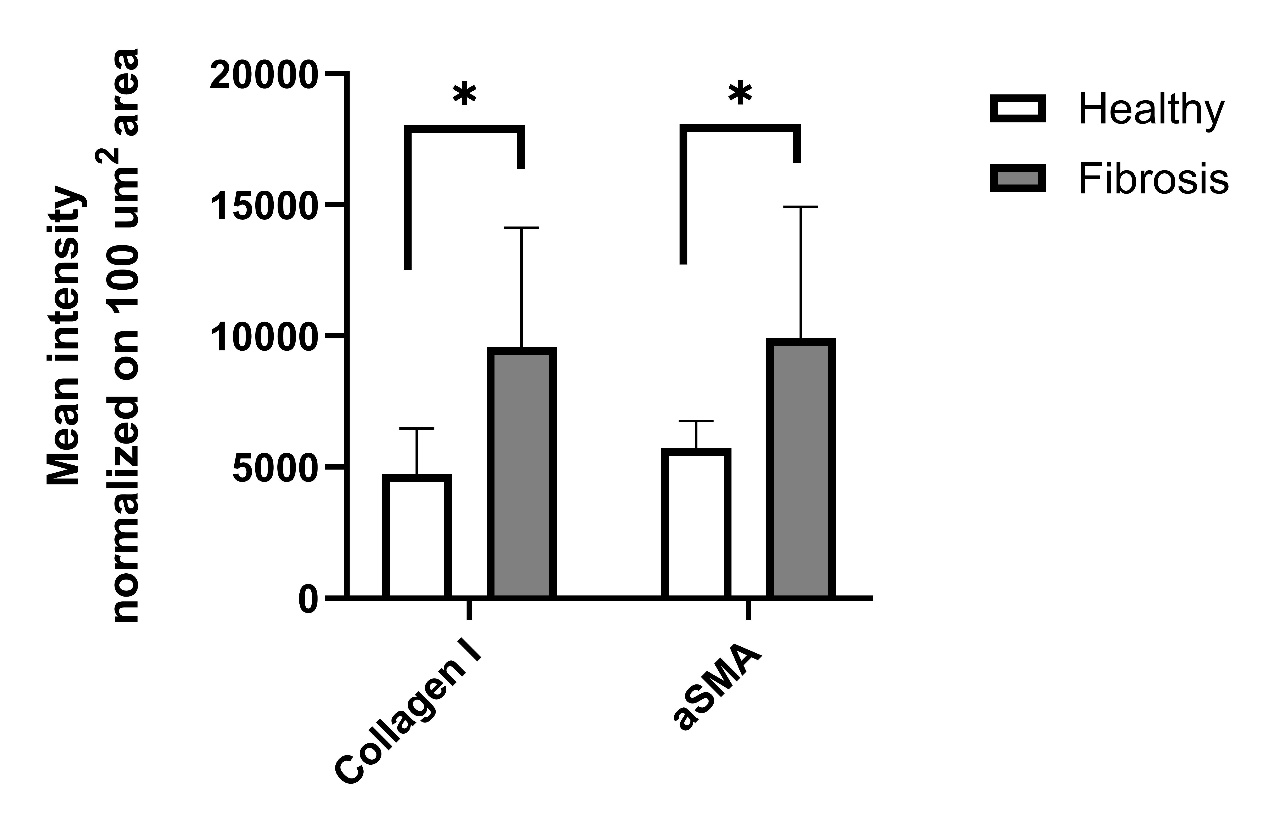


Figure S13. Quantification of normalized mean intensity area of Col I and aSMA expression in healthy (HSMM+ F.Healthy) vs Fibrosis (HSMM+ F.DMD) muscle model based on immunofluorescence images. Unpaired t-test performed to quantify statistical differences. *p<0.05, N=3.


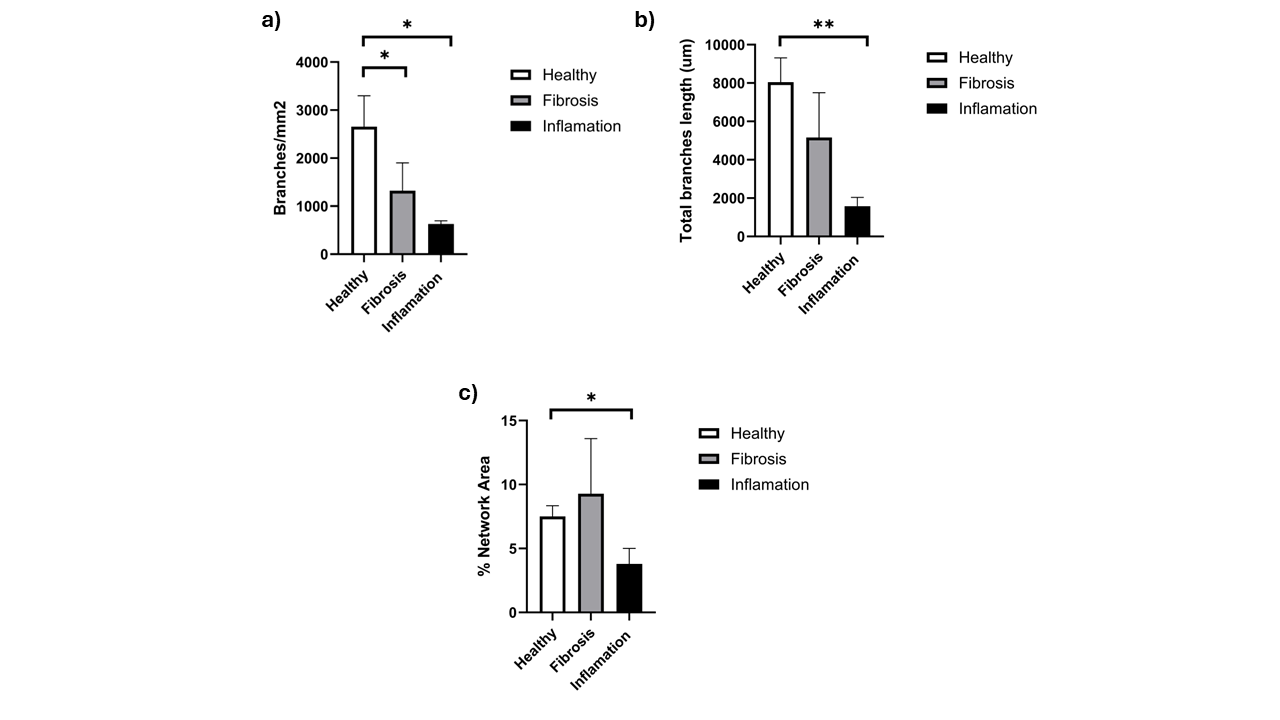


Figure S14. Quantification of GFP+ networks. (a) Branch density (branches/mm^2^), (b) total branch length (µm), and (c) % of GFP+ network area across conditions. Data are shown as mean ± SD; *p < 0.05, **p < 0.01, N=3.


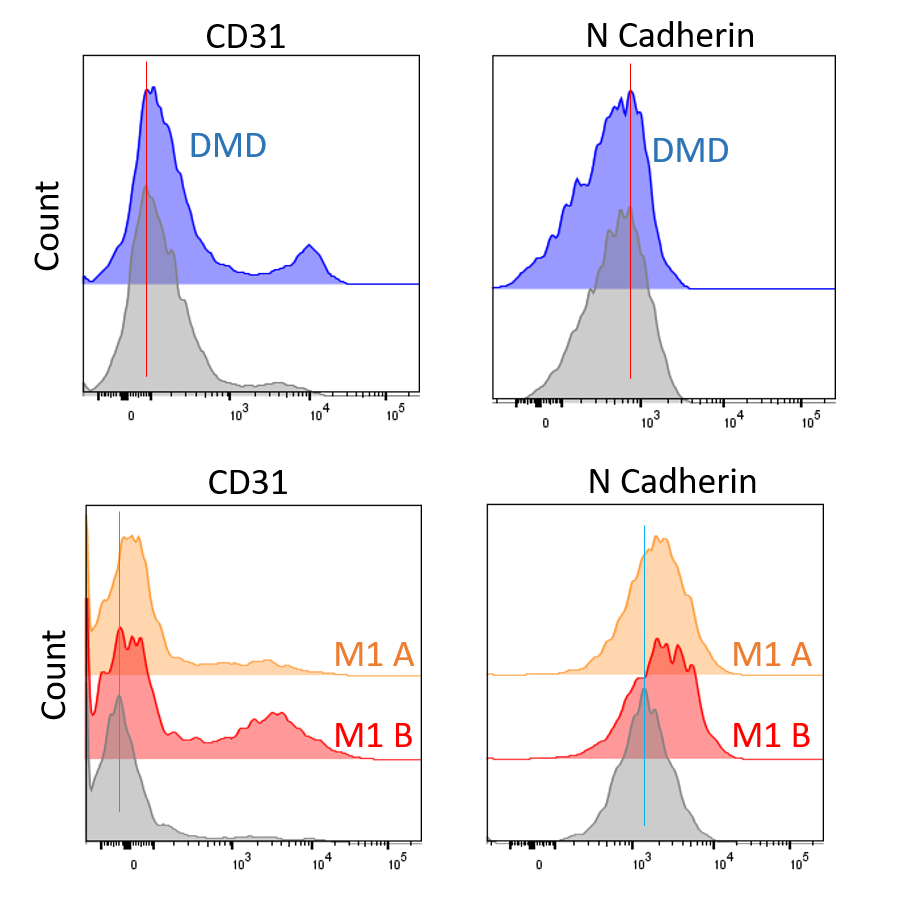


Figure S15. Flow cytometry graphical representation showing positivity of ECs to C31 and N-cadherin in fibrotic DMD (top) and inflamed M1 (bottom) conditions.


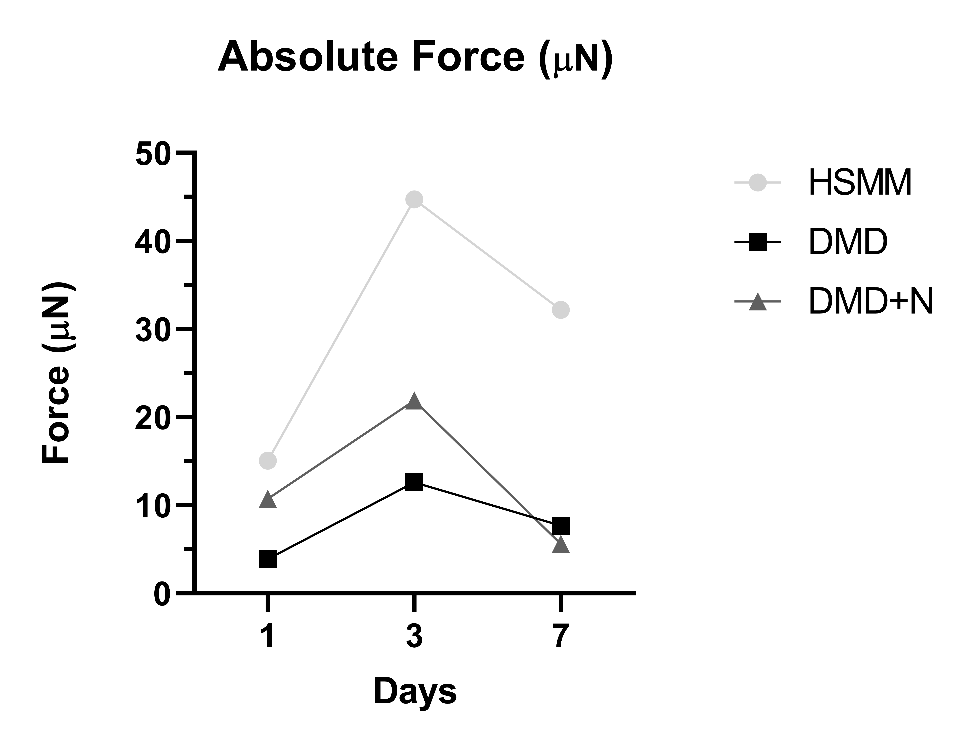


Figure S16. Total contraction force measured from pillar displacement of only muscle (HSMM), fibrotic muscle-fibro myobundles (DMD), and fibrotic muscle-fibro myobundles with Nintedanib (DMD+N).


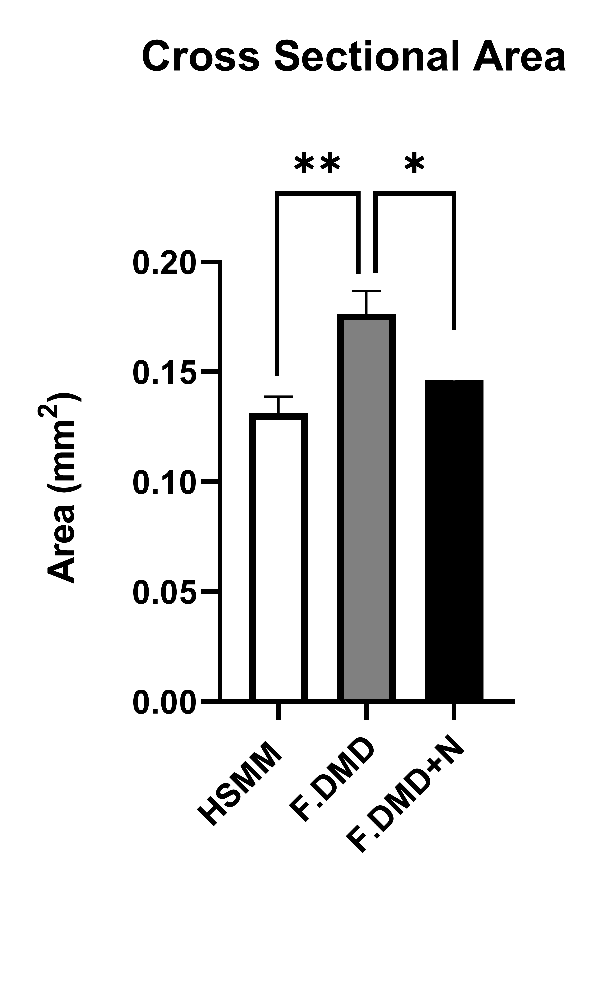


Figure S17. Cross Sectional Area (CSA) of only muscle myobundles (HSMM), fibrotic muscle-fibro myobundles (DMD), and fibrotic muscle-fibro myobundles with Nintedanib (DMD+N).


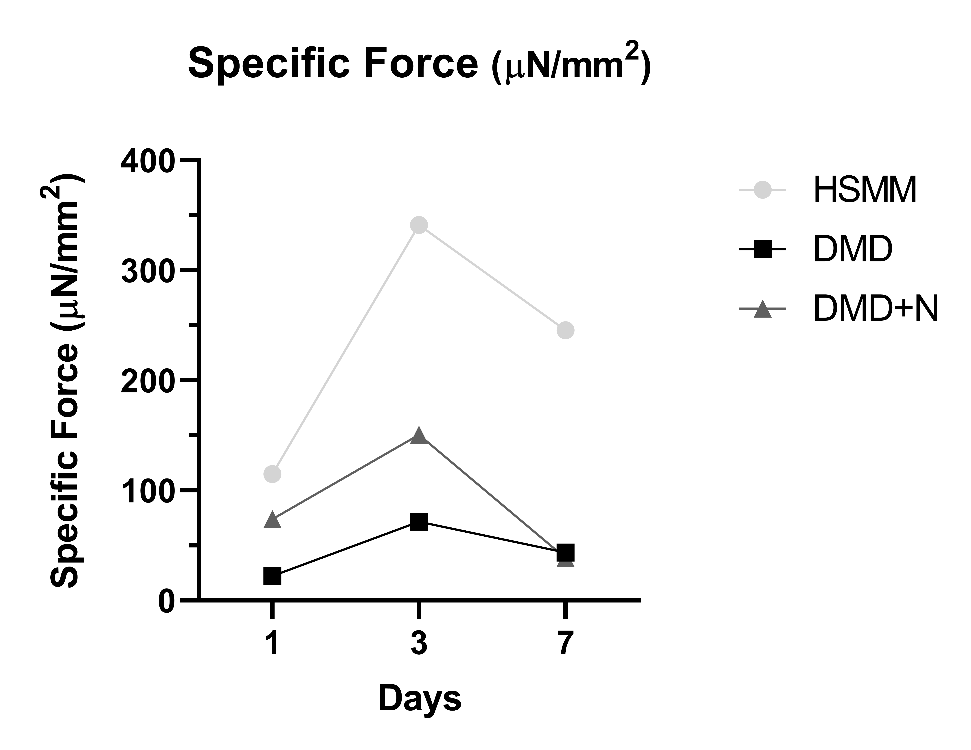


Figure S18. Specific contraction force measured as total contraction force over CSA in each condition: only muscle myobundle (HSMM), fibrotic muscle-fibro myobundles (DMD), and fibrotic muscle-fibro myobundles with Nintedanib (DMD+N).

Table S1. Paraffin embedding process schedule detailing the specific reagents used and the corresponding embedding time for each step.


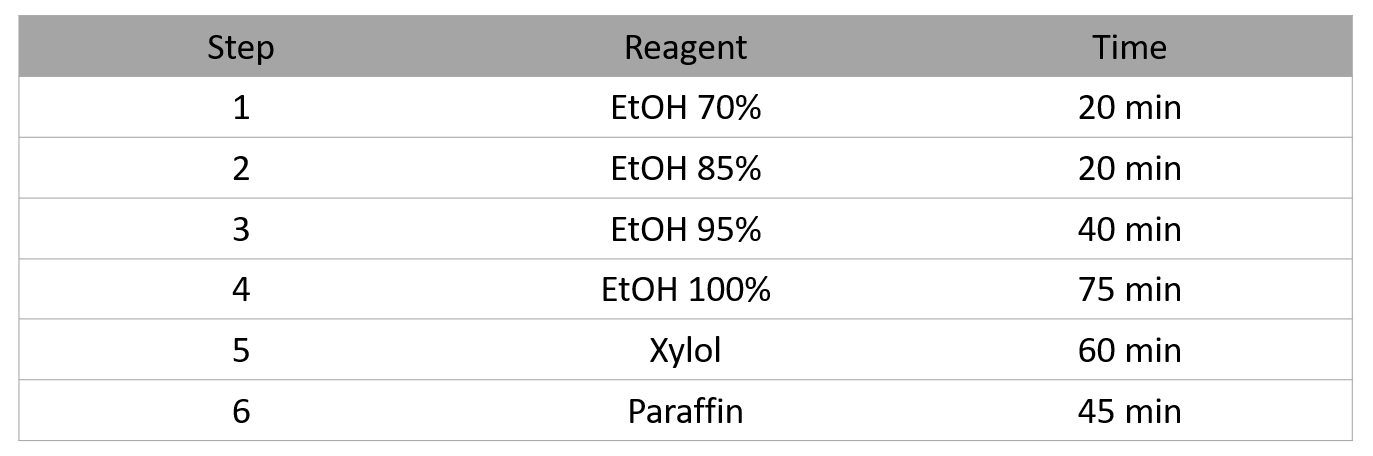


Table S2. De-paraffinization process schedule detailing the specific reagents used and the corresponding embedding time for each step.


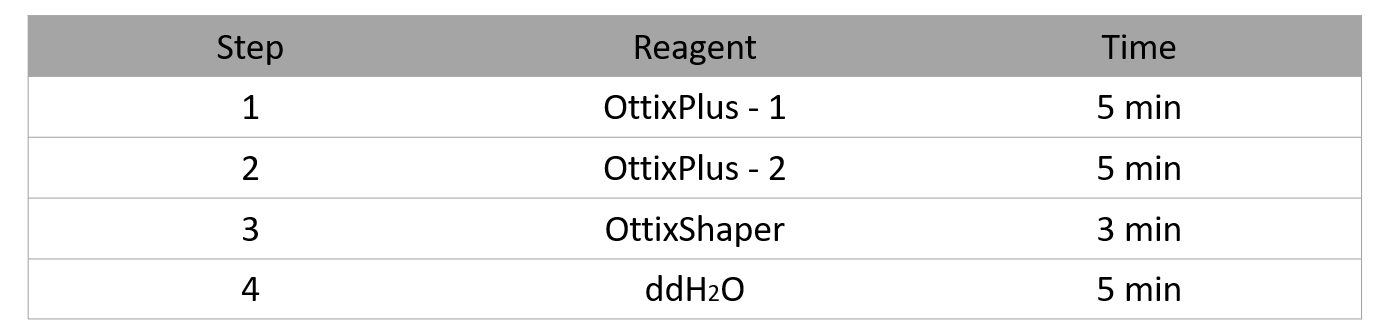


Table S3. List of primary, secondary antibodies and dye used with their specific concentration.


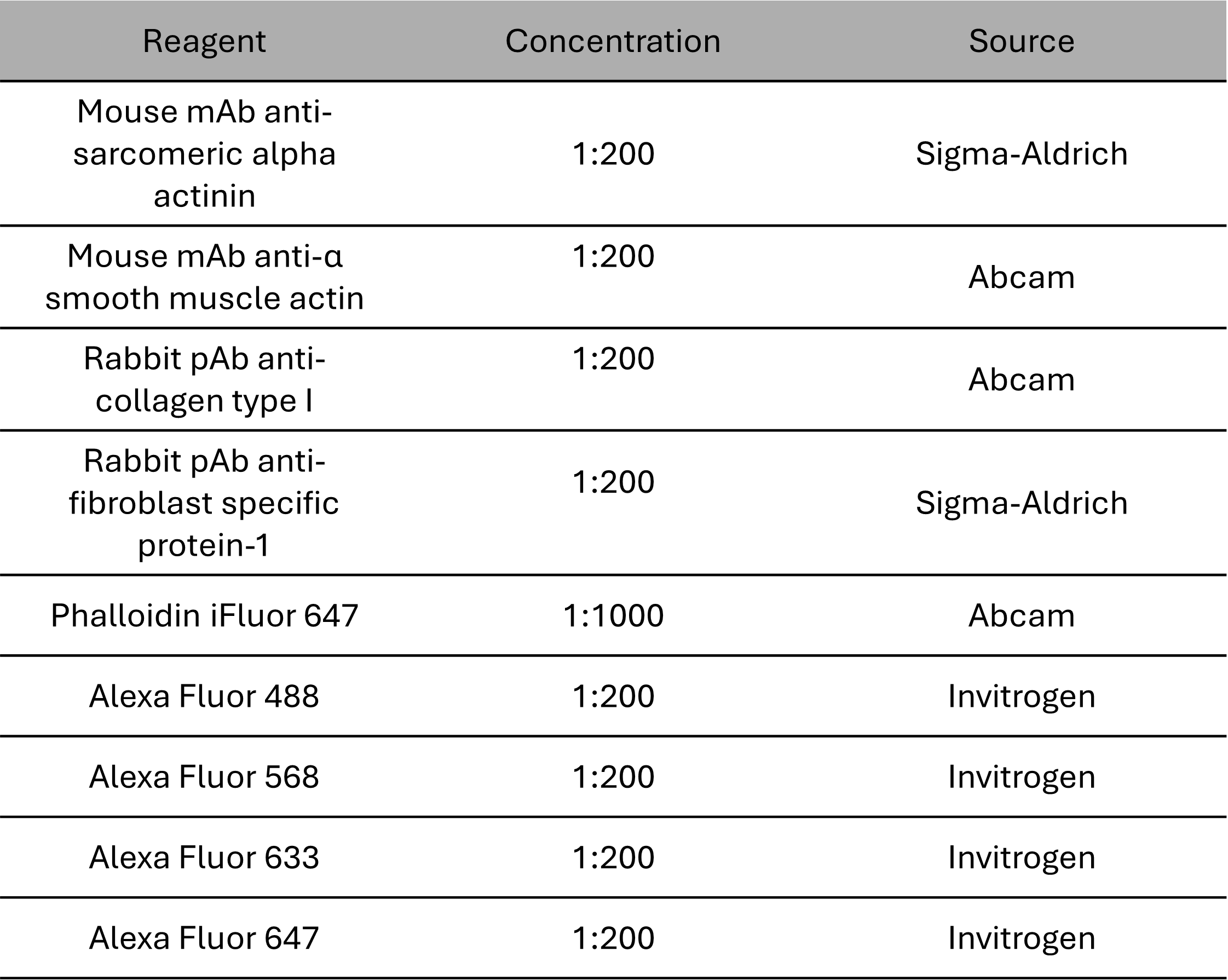


Table S4. Force and height parameters used for computational simulation to predict pillar displacement in different conditions.


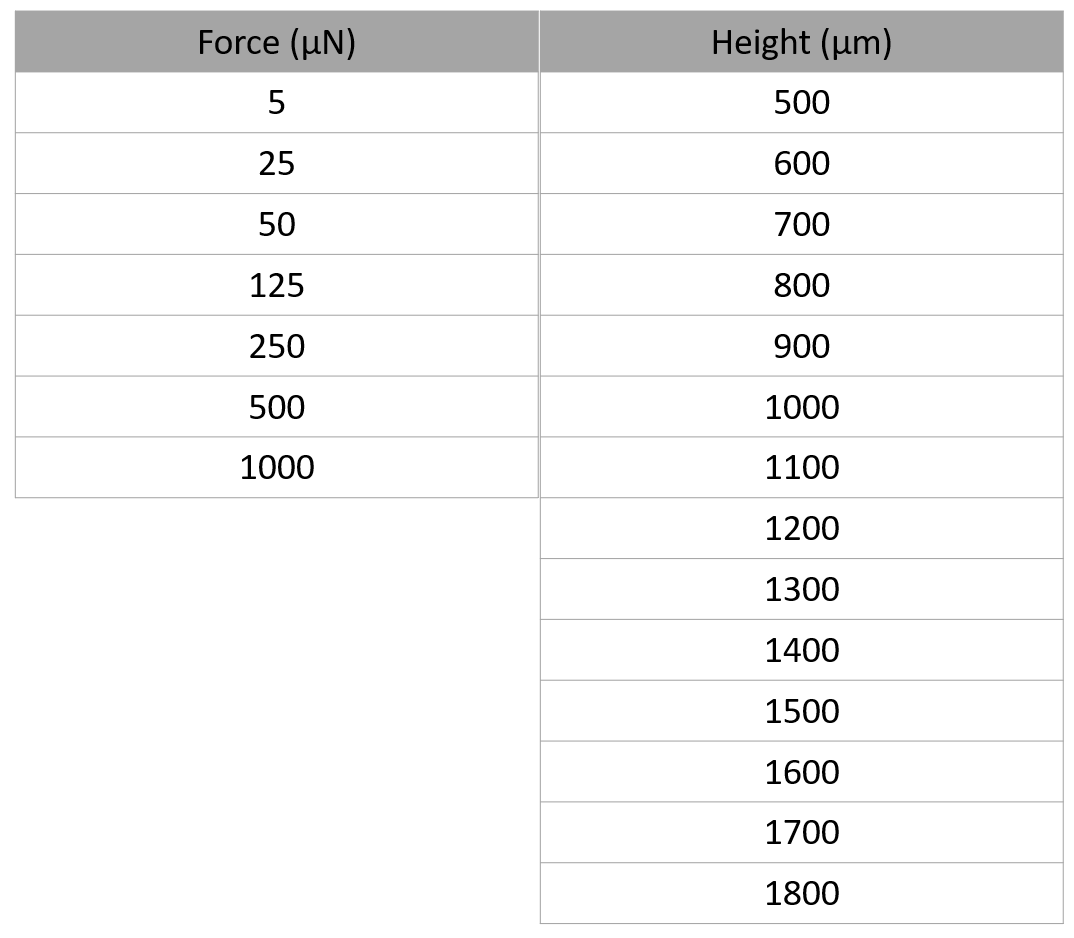


Table S5. Fluorochrome-conjugated antibodies used for flow cytometry.


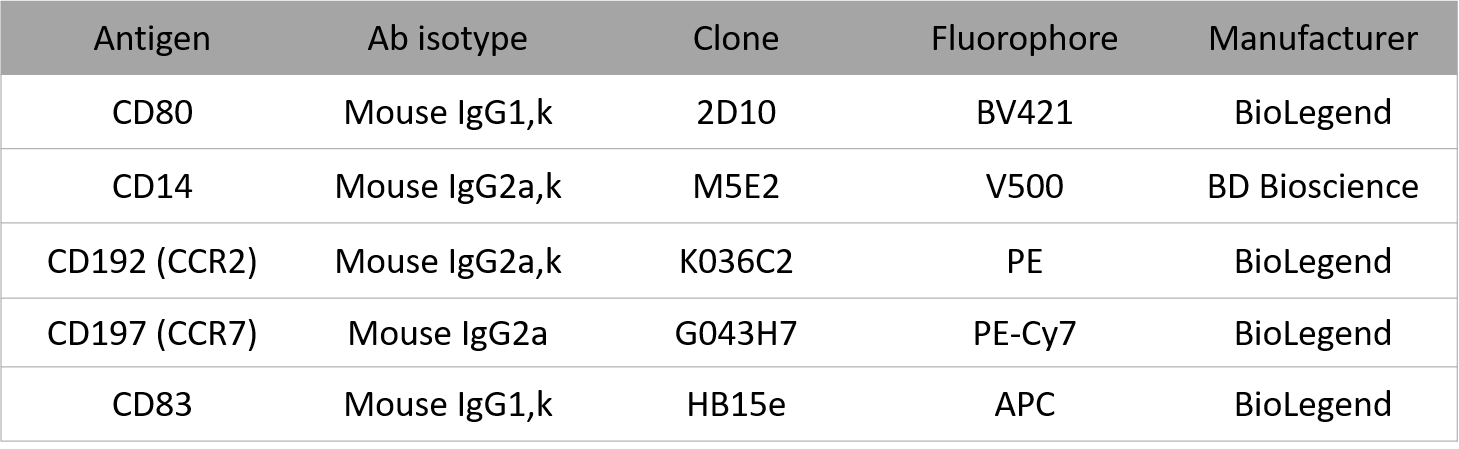


*Video S1. Nanoindentation testing of the 10:0.5 PDMS ratio and 0.5 diameter pillar.*

*Video S2. Nanoindentation testing of the 10:1 PDMS ratio and 0.5 diameter pillar.*

*Video S3. Nanoindentation testing of the 10:0.5 PDMS ratio and 0.75 diameter pillar.*

*Video S4. Nanoindentation testing of the 10:1 PDMS ratio and 0.75 diameter pillar.*

*Video S5. Myobundle contraction upon 1Hz electrical stimulation pattern.*

*Dataset 1. Raw data from Protein Mass Spectrometry.*

*Dataset 2. Elaborated data of protein content for each sample from Protein Mass Spectrometry.*

*Dataset 3. Differentially expressed genes (DEGs) comparing “Day 0 Not Stimulated” vs. “Day 14 Not Stimulated”*

*Dataset 4. Differentially expressed genes (DEGs) comparing “Day 0 Not Stimulated” vs. “Day 14 Stimulated”*

*Dataset 5. Differentially expressed genes (DEGs). Comparison “Stimulated” vs. “Not Stimulated”*

*Dataset 6. Gene set enrichment analysis (GSEA)_Hallmarks. “Stimulated” vs. “Not Stimulated”*
